# A novel prognostic model for hepatocellular carcinoma based on 5 microRNAs related to vascular invasion

**DOI:** 10.1186/s12920-022-01162-7

**Published:** 2022-02-24

**Authors:** Wei Chen, Hao Wang, Tong Li, Te Liu, Wenjing Yang, Anli Jin, Lin Ding, Chunyan Zhang, Baishen Pan, Wei Guo, Beili Wang

**Affiliations:** 1 Department of Laboratory Medicine, Zhongshan Hospital, Fudan University, Shanghai, China; 2 Cancer Center, Shanghai Zhongshan Hospital, Fudan University, Shanghai, China; 3 Department of Laboratory Medicine, Xiamen Branch, Zhongshan Hospital, Fudan University, Xiamen, China; 4 Department of Laboratory Medicine, Wusong Branch, Zhongshan Hospital, Fudan University, Shanghai, China; 5 Shanghai Geriatric Institute of Chinese Medicine, Shanghai University of Traditional Chinese Medicine, Shanghai, China

**Keywords:** Hepatocellular carcinoma, Vascular invasion, microRNA, Overall survival, Prognostic model, Co-expression network

## Abstract

**Background:**

Hepatocellular carcinoma (HCC) is prevalent worldwide with a high mortality rate. Prognosis prediction is crucial for improving HCC patient outcomes, but effective tools are still lacking. Characteristics related to vascular invasion (VI), an important process involved in HCC recurrence and metastasis, may provide ideas on prognosis prediction.

**Methods:**

Tools, including R 4.0.3, Funrich version 3, Cytoscape 3.8.2, STRING 11.5, Venny 2.1.0, and GEPIA 2, were used to perform bioinformatic analyses. The VI-related microRNAs (miRNAs) were identified using Gene Expression Omnibus HCC miRNA dataset GSE67140, containing 81 samples of HCC with VI and 91 samples of HCC without VI. After further evaluated the identified miRNAs based on The Cancer Genome Atlas database, a prognostic model was constructed via Cox regression analysis. The miRNAs in this model were also verified in HCC patients. Moreover, a nomogram was developed by integrating risk score from the prognostic model with clinicopathological parameters. Finally, a potential miRNA-mRNA network related to VI was established through weighted gene co-expression network analysis of HCC mRNA dataset GSE20017, containing 40 samples of HCC with VI and 95 samples of HCC without VI.

**Results:**

A prognostic model of 5 VI-related miRNAs (hsa-miR-126-3p, hsa-miR-148a-3p, hsa-miR-15a-5p, hsa-miR-30a-5p, hsa-miR-199a-5p) was constructed. The area under receiver operating characteristic curve was 0.709 in predicting 5-year survival rate, with a sensitivity of 0.74 and a specificity of 0.63. The nomogram containing risk score could also predict prognosis. Moreover, a VI-related miRNA-mRNA network covering 4 miRNAs and 15 mRNAs was established.

**Conclusion:**

The prognostic model and nomogram might be potential tools in HCC management, and the VI-related miRNA-mRNA network gave insights into how VI was developed.

**Supplementary Information:**

The online version contains supplementary material available at 10.1186/s12920-022-01162-7.

## Introduction

Hepatocellular carcinoma (HCC) is the fourth leading cause of cancer-related deaths and the fifth most common cancer worldwide [1–3]. Although the rapid development of surgery and targeted immunotherapy, the 5-year survival rate of HCC patients was still low [4]. Prognosis prediction is a pivotal point to improve the outcomes of HCC treatment. Many studies revealed that the main reason for the poor prognosis of HCC is post-surgical metastasis and recurrence [5, 6]. Therefore, researchers have analyzed the molecular characteristics of tumor tissues in HCC patients with early recurrence or metastasis to explore prognostic biomarkers or signatures [7, 8]. Some biological processes caused by the tumor occur before recurrence or metastasis. They can be detected by imaging examination, prompting the patient's prognosis more sensitively. Studying the molecular characteristics of these biological processes, such as angiogenesis, can be another strategy for obtaining prognostic signatures [9–12].

Vascular invasion (VI) is one of the biological processes of tumor, and some studies have found that it is a predictor of tumor recurrence and metastasis [13, 14]. With the occurrence of VI, the molecular characteristics of tumor tissues, including microRNAs (miRNAs), have undergone many changes [15, 16]. MiRNAs are short RNA molecules of 19–25 nucleotides, highly conserved among species, regulating gene expression at the post-transcriptional level [17]. Changes in the expression levels of miRNAs can imply the occurrence and development of various diseases, including cancer [18]. Some previous studies have used VI-related miRNAs to construct signatures for assessing the risk of recurrence or predicting the presence of VI in HCC [19, 20]. However, few studies have focused on predicting the overall survival (OS) of HCC patients.

Based on the above, this study was dedicated to constructing a novel prognostic model for HCC patients using VI-related miRNAs. We identified differentially expressed miRNAs between HCC patients with and without VI using data from Gene Expression Omnibus (GEO) database and defined them as VI-related miRNAs. Among them, miRNAs related to prognosis were screened out based on the clinical information from The Cancer Genome Atlas (TCGA) database and utilized to construct a prognostic model and nomogram. The miRNAs in this model were further validated with patient tissues. In addition, we also constructed a miRNA-mRNA network related to VI, which might provide new ideas on the VI-targeted HCC treatment.

## Materials and methods

### Clinical samples

A total of 23 HCC patients in Zhongshan Hospital Affiliated to Fudan University, who underwent resection without preoperative anti-tumor treatments, were enrolled in our study. Five of them had VI, and 18 did not have VI. Their tumor samples were collected in surgery and used to verify the expression levels of miRNAs identified in the following analyses by real-time polymerase chain reaction (PCR). All patients enrolled in this study provided written informed consent, and their clinical information was shown in Additional file 1: Table S1.

### Data retrieval

Our data were obtained from GEO database (https://www.ncbi.nlm.nih.gov/geo/) and TCGA data portal (https://portal.gdc.cancer.gov/). The keywords used to search for GEO datasets were “((Hepatocellular carcinoma) AND vascular invasion) AND "Homo sapiens"” plus “Non-coding RNA profiling by array” for miRNA or plus “Expression profiling by array” for mRNA. The datasets were filtered according to the following criteria: (1) there were no restrictions on the etiology of HCC patients; (2) containing VI information for patients; (3) sample size of each group ≥ 40. We finally selected datasets GSE67140 and GSE20017 for subsequent analyses [14, 21]. The miRNA dataset GSE67140 (platform: GPL8786 [miRNA-1] Affymetrix Multispecies miRNA-1 Array) contains 81 samples of HCC with VI and 91 samples of HCC without VI. The mRNA dataset GSE20017 (platform: GPL8432 Illumina HumanRef-8 WG-DASL v3.0) contains 40 samples of HCC with VI and 95 samples of HCC without VI. In the TCGA data portal, we downloaded the miRNA isoform expression quantification data (Illumina HiSeq miRNA-Seq platform) of HCC patients and the corresponding clinical information on December 15, 2020. There were 373 patients in total, and their clinical information was shown in Additional file 1: Table S2.

### Identification of VI-related miRNAs and mRNAs

We used the normalizeBetweenArrays method to normalize the expression matrix of dataset GSE67140 and used the "limma" R package to perform differential expression analysis [22]. With the standard of adj.*P*.Val < 0.05 and |log^2^ fold change (FC)|> 2, we got the VI-related miRNAs. Then, by comparing the nucleotide sequence with mature miRNAs, the mature miRNA names were obtained.

The volcano maps and heatmaps of differentially expressed miRNAs in samples of HCC patients with VI compared with patients without VI were plotted using "ggplot2" R package and "pheatmap" R package, respectively.

### GO and KEGG analysis

We used Funrich version 3 software to perform Gene Ontology (GO) and Kyoto Encyclopedia of Genes and Genomes (KEGG) functional enrichment based on upregulated or downregulated VI-related miRNAs.

### Survival analysis

The prognostic information was also provided by TCGA. We used the “sur.cut” function of the "survival" R package to find the best cut-off value of each miRNA. Then we used Stata 16.0 to perform Kaplan–Meier (K–M) analyses and plot survival curves. Log-rank tests were performed, and log-rank.*P* < 0.01 was considered statistically significant.

### Construction of prognostic model and prognostic nomogram

We used univariate Cox regression analysis, also called proportional hazard regression analysis, to evaluate the prognostic value of identified miRNAs in survival analysis. *P* < 0.05 was considered statistically significant. Then we selected the optimal miRNA combination based on the minimum Akaike information criterion (AIC) value to establish a prognostic model. Multivariate Cox regression analysis was used to obtain regression coefficients (β). The “predict (cox, type = "risk")” function was used in the R software to calculate the risk score of each patient. The calculation formula was as follows: Risk Score = ${\widehat{h}}_{i}\left(t\right)={\widehat{h}}_{0}(t)\mathrm{exp}({{x}^{^{\prime}}}_{i}\widehat{\beta })$. The expression of miRNAs was processed by log2 transformation. According to the median risk score, the patients were divided into high-risk and low-risk groups. In order to assess the prognostic prediction ability of the prognostic model, we plotted a K–M survival curve and a time-dependent receiver operating characteristic (tdROC) curve. Univariate and multivariate Cox regression analyses were used to estimate whether risk score is an independent (*P* < 0.05) prognostic risk factor. We also used multi-index tdROC curves to compare the prognostic efficacy of risk score and other clinicopathological parameters. The prognostic efficacy of each parameter was evaluated by calculating the area under the curve (AUC) [23, 24]. The tdROC curves in this study were based on 5-year survival rate.

We constructed a nomogram by combining traditional clinical parameters such as age, gender, T-stage, and AJCC stage with risk score to predict the 1-, 3-, and 5-year OS of HCC patients. The construction and verification of the nomogram were carried out using the “rms” R package.

### Evaluation of the prognostic model

The HCC patients in TCGA were classified into high-risk and low-risk groups based on the median risk sore. Stata 16.0 was used to perform K-M analysis and plot survival curve. The ROC curve was applied to assess the sensitivity and specificity in predicting prognosis and compare the efficacy with other clinicopathological parameters. Scatter plot and heatmap were plotted to exhibit the difference in survival status between the high-risk and low-risk groups. Besides, univariate and multivariate Cox regression analyses were performed to assess whether the risk score in prognostic model was an independent prognostic factor of HCC patients.

### Real-time PCR

TRIzol reagent (Invitrogen, Carlsbad, CA, USA) was used to extract RNA from patient tissues. MiRNAs in the extracted samples were reverse-transcribed and quantified using All-in-One™ miRNA qPCR Kit (GeneCopoeia, Rockville, Md, USA). The real-time PCR program was performed according to the GeneCopoeia kit instructions. U6 snRNA was considered as the internal control. The All-in-One™ miRNA qPCR Primers HmiRQP0223, HmiRQP0391, HmiRQP0099, HmiRQP0204, and HmiRQP0290 (GeneCopoeia, Rockville, Md, USA) were used to detect hsa-miR-15a-5p, hsa-miR-30a-5p, hsa-miR-126-3p, hsa-miR-148a-3p, and hsa-miR-199a-5p, respectively. The relative miRNA expression level was calculated using 2^−△Ct^. Student’s t-test was used to determine the difference between two groups.

### Establishment of miRNA-mRNA network

The 5 miRNA target genes were predicted by the miRWalk online tool (http://mirwalk.umm.uni-heidelberg.de/). Weighted gene co-expression network analysis (WGCNA) algorithm is a useful statistical method for constructing co-expression networks with similar biological functions [25, 26]. The matrix of dataset GSE20017 was used to construct stable co-expression modules through the “WGCNA” R package [25, 26]. VI-related mRNAs are defined in these modules. The reads with a value less than 0 in the matrix were revised to 0. The intersection of predicted targets and VI-related mRNAs was found using Venny 2.1.0 online tool to discover the potential miRNA-mRNA mechanisms (https://bioinfogp.cnb.csic.es/tools/venny/index.html). Then the potential miRNA-mRNA network associated with the occurrence of VI was visualized by Cytoscape 3.8.2 software [27]. Furthermore, STRING 11.5 online tool (https://cn.string-db.org/) was used to find protein–protein interactions (PPIs). In addition, GEPIA 2 online tool was used to verify the correlation between these hub genes and clinical staging (http://gepia2.cancer-pku.cn/#analysis) [28].

#### Statistical analysis

The bioinformatics packages were run by the R 4.0.3 software, and the criteria for statistical significance were mentioned above. The P values of differential expression analysis were corrected by the Benjamini–Hochberg method to obtain adj.*P*.Val or FDR. In addition, the Chi-square test was used to compare survival status between the high-risk and low-risk groups based on the prognostic model. At the same time, F test was used to compare the variances. When the variances were uneven (*P* < 0.1), Welch’s t-test was used. *P*-value < 0.05 was considered statistically significant. The statistical software was R 4.0.3, Stata 16.0, and GraphPad Prism 7.

## Results

### Identification of VI-related miRNAs in HCC and their functional enrichment analysis

**Fig. 1:**
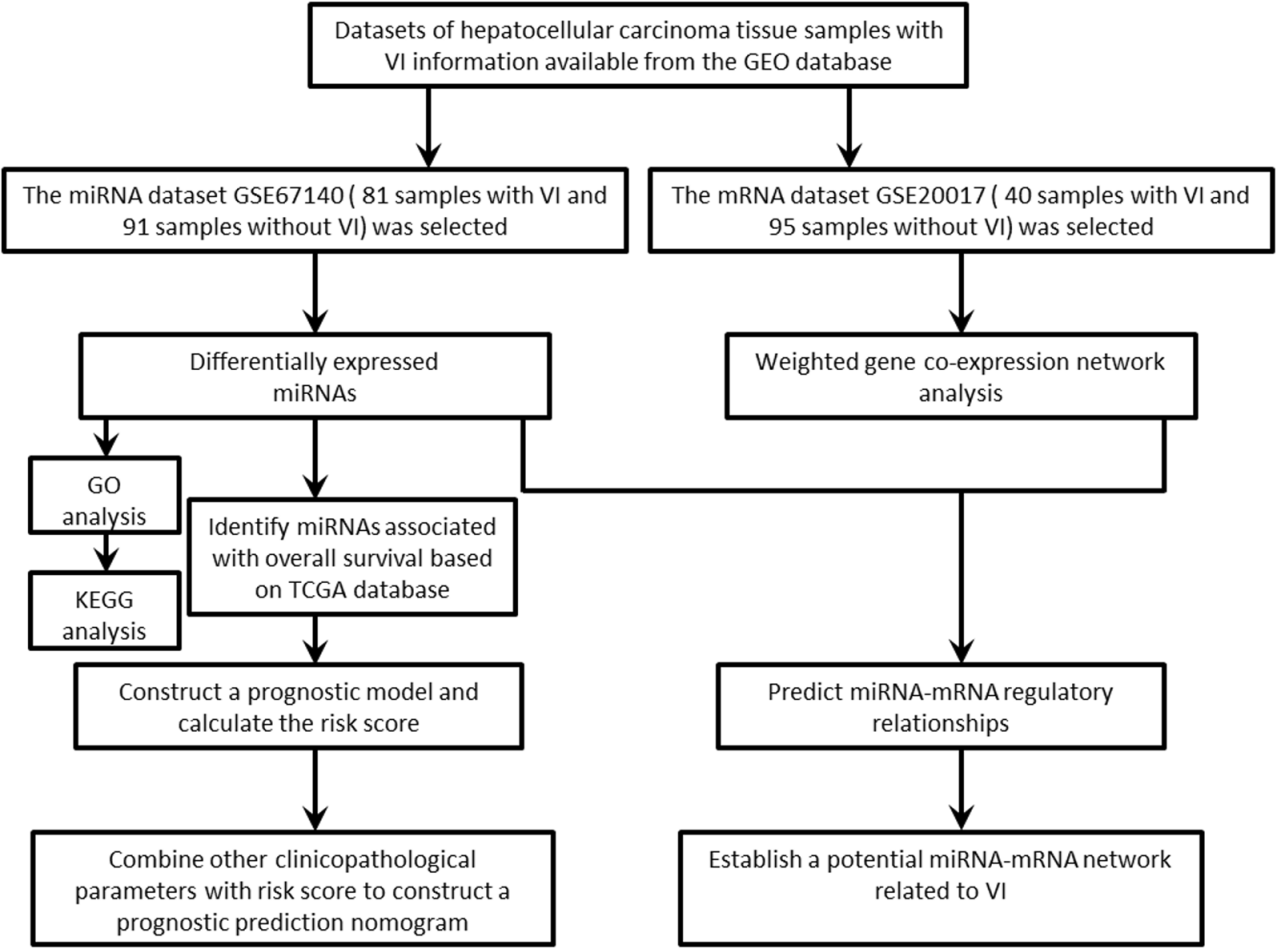
The workflow of the analysis process

**Fig. 2:**
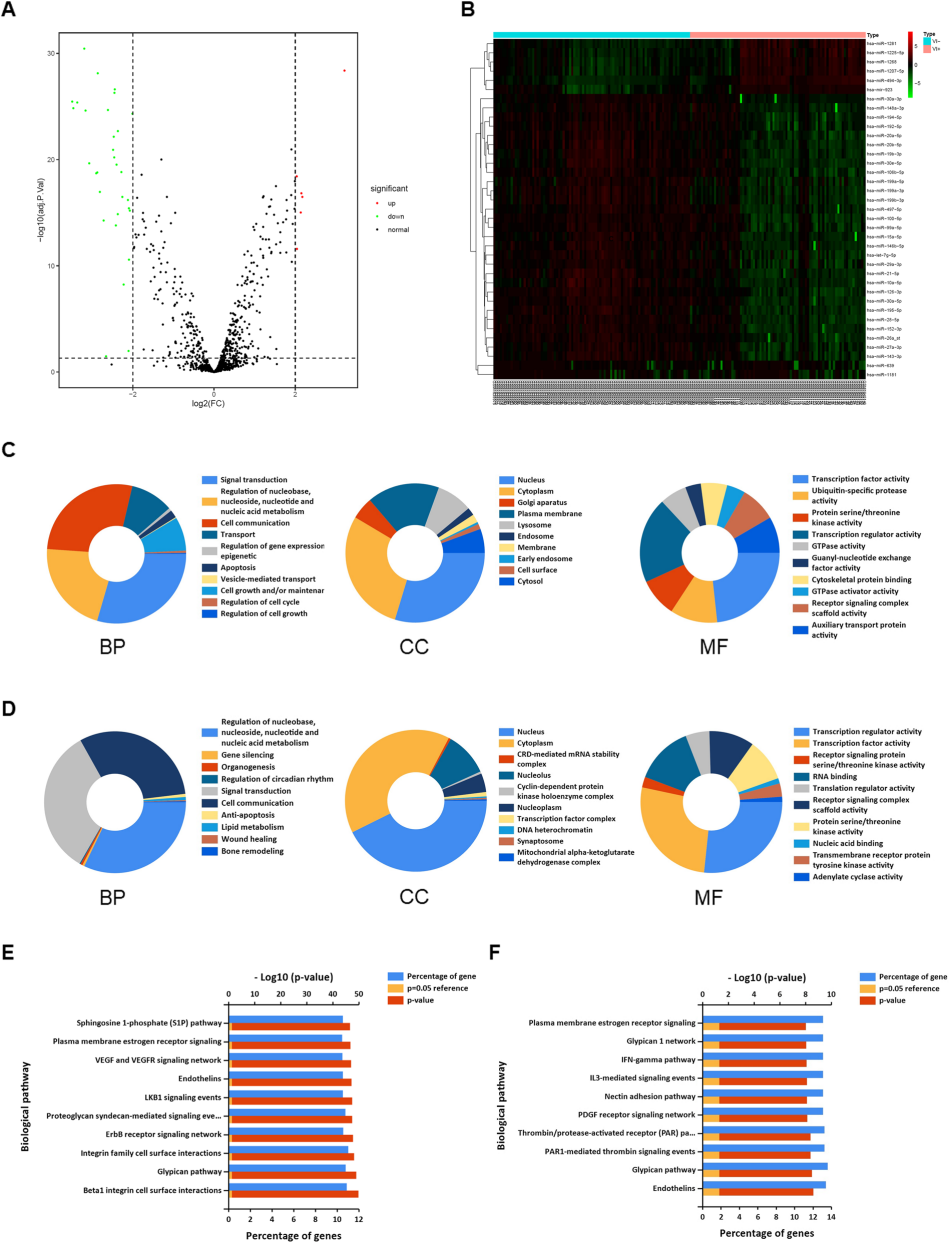
Identification of VI-related miRNAs in HCC and their functional enrichment analysis. Volcano map (**a**) and heatmap (**b**) of aberrantly expressed miRNAs between HCC samples with and without VI. GO analysis results showed the enriched biological processes, cell components, and molecular functions potentially associated with 31 downregulated miRNAs (**c**) and 6 upregulated miRNAs **(d)**. KEGG analysis results showed the pathways potentially associated with 31 downregulated miRNAs (**e**) and 6 upregulated miRNAs (**f**)

As shown in the flow chart (Fig. 1), we retrieved the dataset GSE67140 in GEO database according to the inclusion criteria. A total of 31 downregulated and 6 upregulated miRNAs were found in HCC with VI group by differential expression analysis and defined as VI-related miRNAs (Fig. 2a, b).

The downregulated or upregulated miRNAs were used to perform GO and KEGG analyses. Downregulated miRNAs were chiefly enriched in GO terms related to signal transduction (biological process, BP); nucleus (cell component, CC); transcription factor activity (molecular function, MF) (Fig. 2c). Upregulated miRNAs were chiefly enriched in GO terms related to regulation of nucleobase, nucleoside, nucleotide, and nucleic acid metabolism (BP); nucleus (CC); transcription regulation activity (MF) (Fig. 2d). KEGG analysis showed that downregulated miRNAs were chiefly enriched in beta1 integrin cell surface interactions, glypican pathway and integrin family cell surface interactions (Fig. 2e), while upregulated miRNAs were chiefly enriched in endothelins, glypican pathway and *PAR1*-mediated thrombin signaling events (Fig. 2f).

### Selection of miRNAs related to HCC patients’ OS

**Fig. 3:**
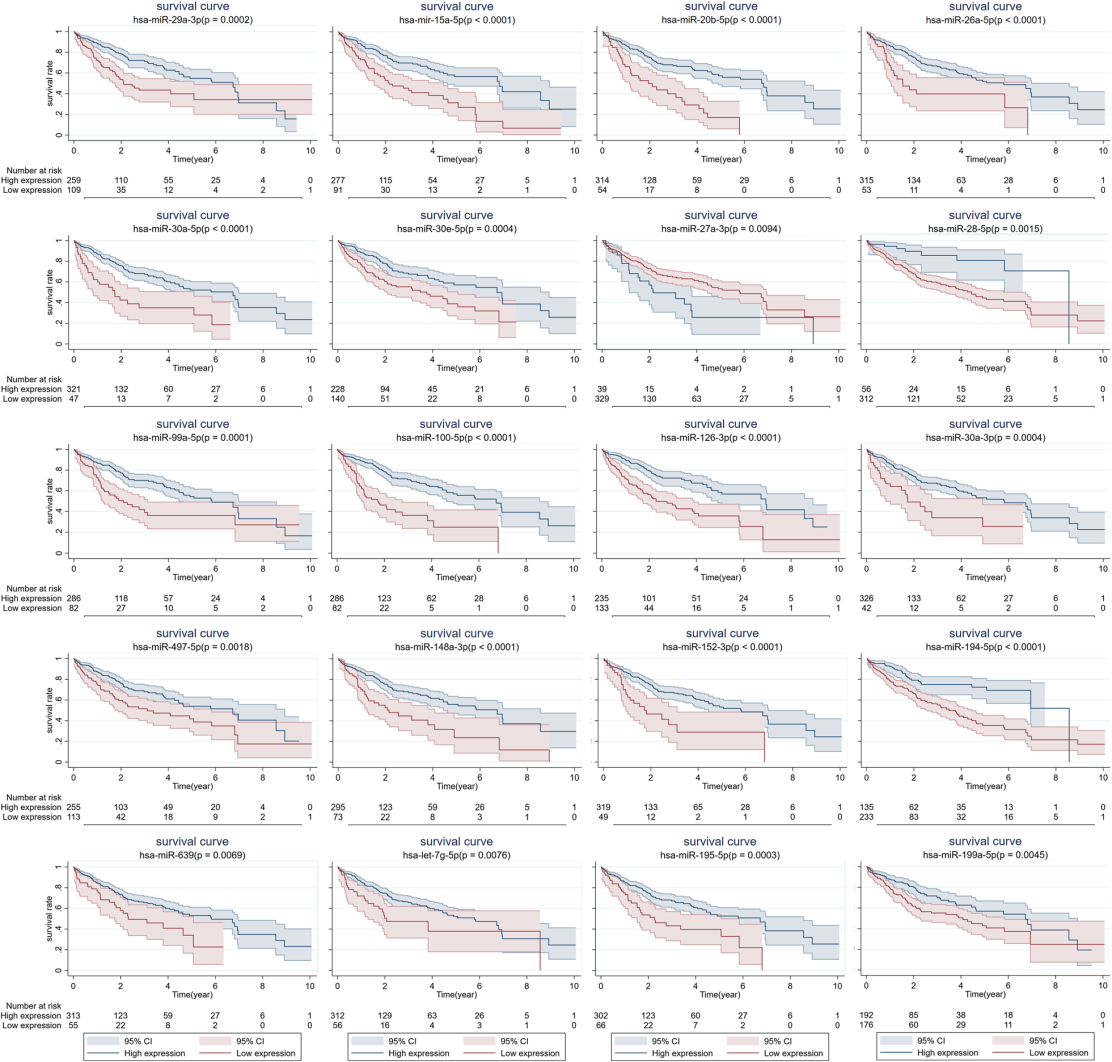
The survival analyses of HCC patients stratified by different expression levels of miRNAs. Kaplan–Meier survival curves indicated that there were 20 miRNAs significantly correlated with patients’ overall survival (OS) based on TCGA database

Using the miRNA isoform expression quantification data and the corresponding clinical prognostic information from TCGA, we plotted the survival curves for all the 37 VI-related miRNAs to evaluate their prognostic value. Patients were divided into two groups with high or low expression based on the best cut-off value of each miRNA expression. The results showed that 20 out of 37 miRNAs were significantly related to the patients’ OS. That is, these 20 miRNAs had prognostic value to some extent (Fig. 3; Additional file 1: Table S3). A total of 373 samples were included in the survival analysis, and 5 of them had no postoperative follow-up information.

### Construction and validation of the prognostic model in HCC patients

**Fig. 4:**
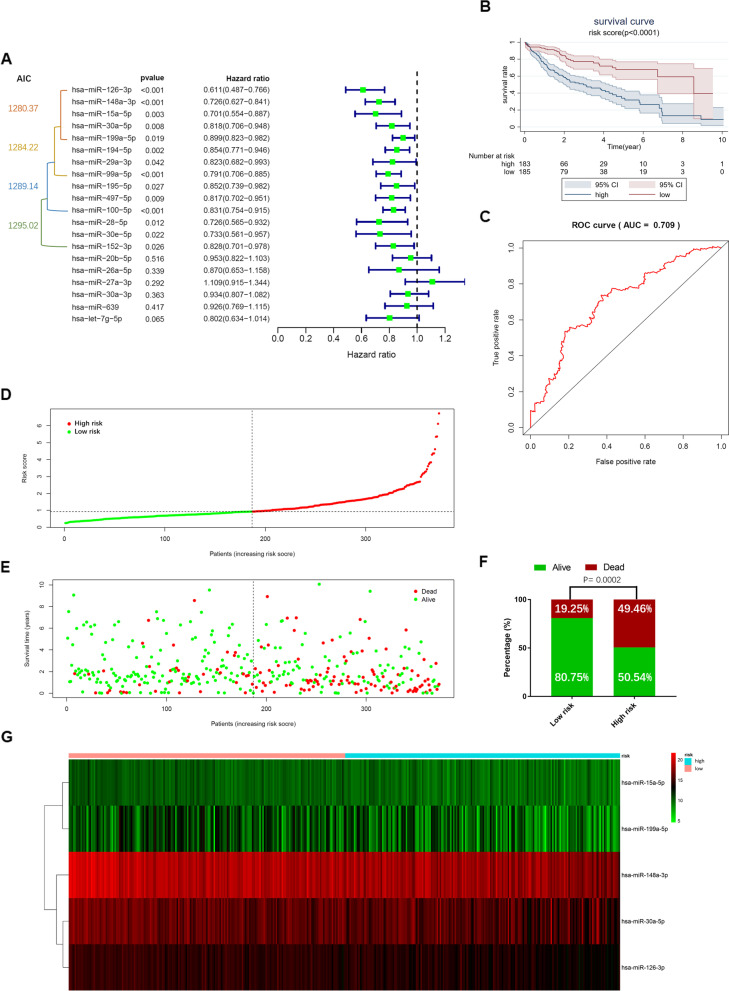
Construction and validation of the prognostic model in HCC patients. (**a**) The univariate Cox regression analysis and Akaike information criterion (AIC) determined 5 VI-related miRNAs to build prognostic model, namely hsa-miR-126-3p, hsa-miR-148a-3p, hsa-miR-15a-5p, hsa-miR-30a-5p, hsa-miR-199a-5p. **(b**) Kaplan–Meier survival analysis showed that the survival time of patients with high-risk scores is significantly shorter than those with low-risk scores. The risk scores were calculated based on the 5-miRNA prognostic model. (**c**) Receiver operating characteristic (ROC) curve showed that the prognostic model has good accuracy in predicting 5-year overall survival of HCC patients from the TCGA database. (**d**) Distribution of risk scores of high- and low-risk HCC patients. Scatter plot (**e**) and bar plot (**f**) represented the correlation between survival time or survival state and risk score of HCC patients based on the prognostic model. (**g**) Heatmap showed that high-risk patients expressed lower levels of each miRNA biomarker

Then we performed univariate Cox regression analysis on these 20 miRNAs and found 14 ones that could significantly indicate prognosis. Finally, a total of 5 miRNA biomarkers (hsa-miR-126-3p, hsa-miR-148a-3p, hsa-miR-15a-5p, hsa-miR-30a-5p, hsa-miR-199a-5p) were determined based on the minimum AIC value to jointly construct a prognostic model (Fig. 4a). Based on this prognostic model, we calculated the risk score of each patient (Additional file 1: Table S4).

In order to evaluate the performance of the prognostic model, we divided the patients into high-risk and low-risk groups based on the median of risk scores (186 cases in the high-risk group, 187 cases in the low-risk group). The results of K–M analysis showed that the high-risk group had a significantly worse prognosis than the low-risk group (*P* < 0.0001). The 3-year survival rates of the high-risk and low-risk groups were 49.7% and 77.2%, respectively, while the 5-year survival rates were 34.0% and 67.7%, respectively (Fig. 4b). The AUC value of the tdROC curve was 0.709, with a sensitivity of 0.74 and a specificity of 0.63, indicating that the prognostic model had satisfactory prognostic prediction power (Fig. 4c). The scatter plots showed the distribution of patients' risk score (Fig. 4d) and the correlation between survival time and risk score (Fig. 4e). The bar plot showed a significant difference in survival status between high-risk and low-risk groups (*P* = 0.0002). Surviving patients in the high-risk group were significantly fewer than in the low-risk group (Fig. 4f). Heatmap showed that high-risk patients expressed lower levels of each miRNA biomarker, indicating that these 5 miRNAs were all protective factors (Fig. 4g).

### The independent prognostic role of risk score in HCC

**Fig. 5:**
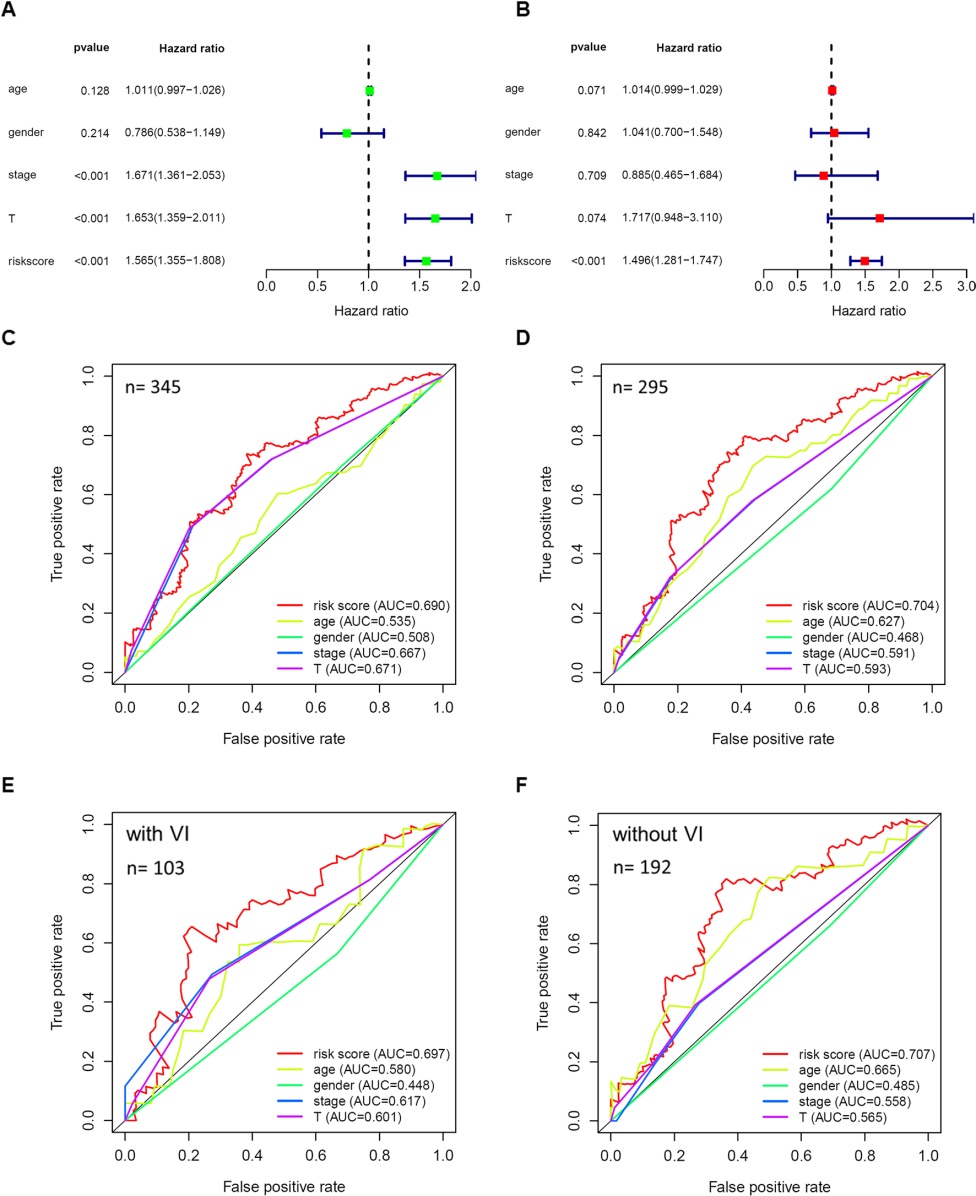
The independent prognostic role of risk score in HCC. Forrest plots of the univariate (**a**) and multivariate (**b**) Cox regression analyses in HCC indicated that risk score might be an independent prognostic factor. The ROC curves showed the performance of the risk score and other clinicopathological parameters (age, gender, AJCC stage, and T-stage) in predicting 5-year overall survival of HCC patients (**c**) and patients with VI information available (**d**). (**e, f**) The ROC curves for two subgroups of patients in D

In order to explore whether the risk score is an independent prognostic risk factor, multiple clinicopathological parameters (age, gender, AJCC stage, and T-stage) and risk score were analyzed by univariate and multivariate Cox regression analyses. The result of univariate Cox regression analysis showed that AJCC stage, T-stage, and risk score were valuable prognostic factors. At the same time, there was no significant difference in prognosis among patients of different ages and genders (Fig. 5a). The result of multivariate Cox regression analysis suggested that risk score might be an independent prognostic risk factor of HCC patients (Fig. 5b).

We used the multi-index tdROC curve to compare the accuracy of different clinicopathological parameters and risk scores in predicting the 5-year OS in HCC patients. Among all patients with these clinicopathological parameters (n = 345), the AUC values of risk score, T-stage, and AJCC stage were 0.690, 0.671, and 0.667, respectively (Fig. 5c). Furthermore, a similar result was obtained in patients with VI information (n = 295), and The AUC values of the three predictors were 0.704, 0.593, and 0.591, respectively (Fig. 5d). Then we divided the patients in Fig. 5d into HCC with VI group (n = 103) and HCC without VI group (n = 192) according to the existence of VI and found that the AUC value of risk score in both groups was higher than those of other clinicopathological parameters. Moreover, in the group without VI, the risk score showed the greatest prognostic advantage over the classic cancer staging system, T-stage or AJCC stage (AUC: 0.707 vs. 0.565 or 0.558) (Fig. 5e, f). To conclude, risk score was demonstrated to be a valuable prognostic risk indicator for HCC patients.

### Construction and validation of the prognostic nomogram with risk score as one of the parameters

**Fig. 6:**
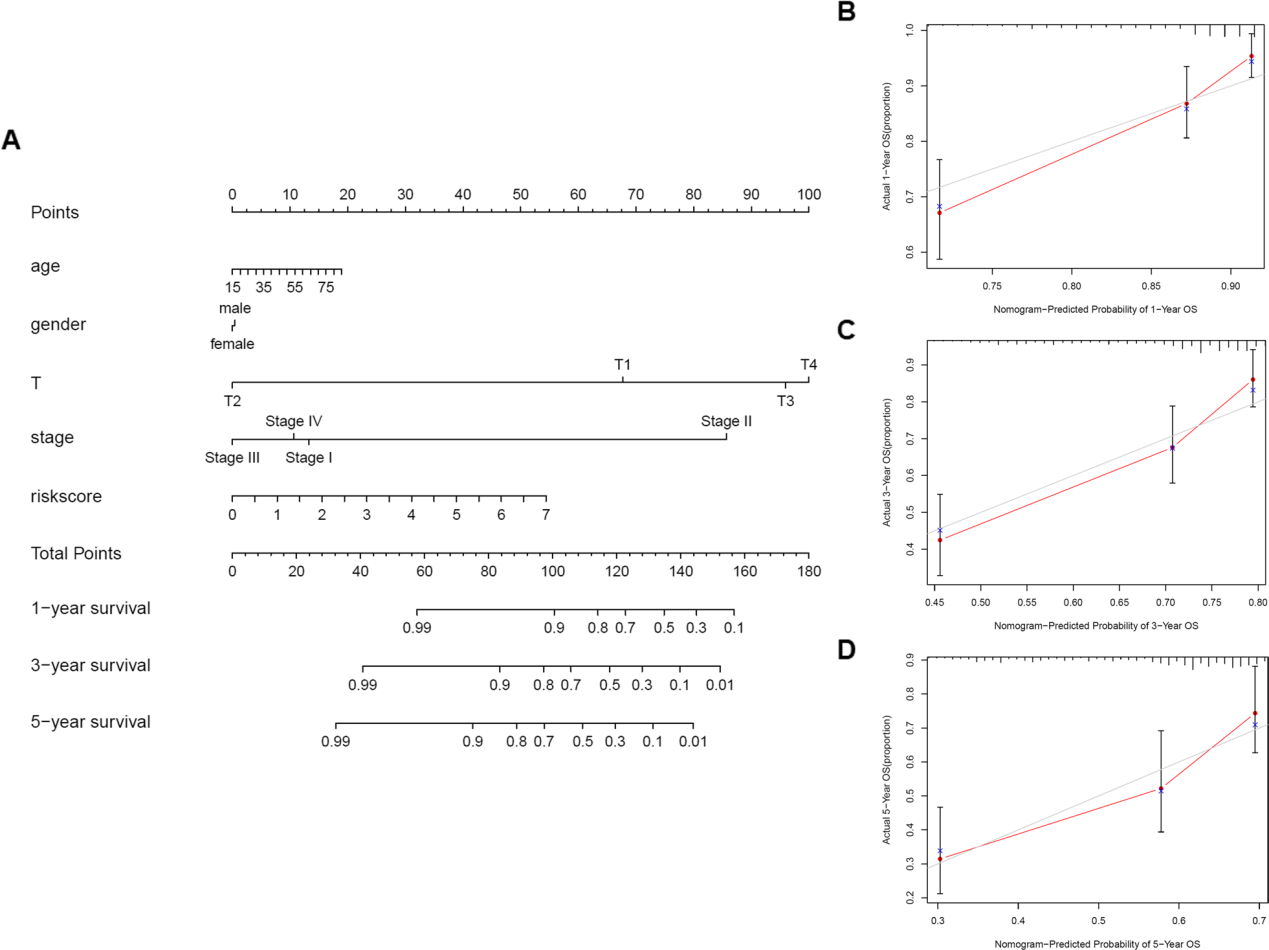
Construction and validation of the prognostic nomogram with risk score as one of the parameters. (**a**) The nomogram used age, gender, T-stage, AJCC stage, and risk score to predict the 1-, 3-, and 5-year OS of HCC patients. The calibration plot to evaluate the concordance between the predicted and actual 1-year (**b**), 3-year (**c**), and 5-year (**d**) survival rates

For better evaluating the patients’ prognosis, we combined multiple clinicopathological parameters (age, gender, T-stage, AJCC stage) and risk score to construct a prognostic nomogram to comprehensively evaluate the patient’s prognosis and predict the patients’ 1 year, 3-year, and 5-year survival rates (Fig. 6a). We also used calibration plots to evaluate the concordance between the nomogram-predicted and actual 1-year, 3-year, and 5-year survival rates. The predicted lines were consistent with the reference lines (Fig. 6b–d). These results indicated that the prognostic nomogram was credible.

### The experimental verification of miRNA expression in HCC samples

**Fig. 7:**
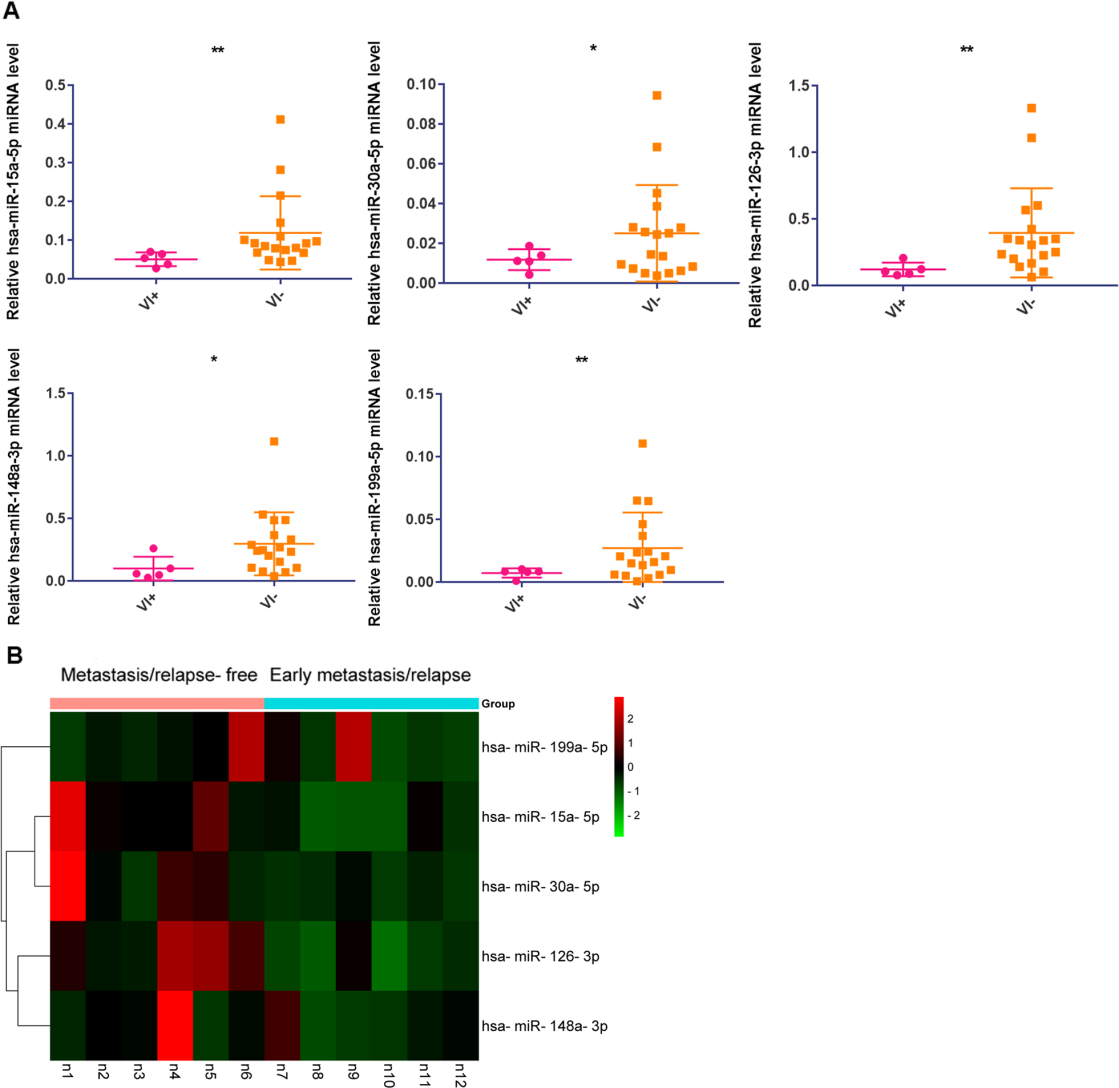
Real-time PCR validation of the expression levels of the 5 miRNAs in patient tissues. (**a**) hsa-miR-15a-5p, hsa-miR-30a-5p, hsa-miR-126-3p, hsa-miR-148a-3p, and hsa-miR-199a-5p were significantly downregulated in tissue samples from HCC patients with VI. (**b**) In HCC patients without VI, these 5 miRNAs' expression level was lower in patients with early metastasis/relapse

We further verified the expression levels of the 5 miRNAs (hsa-miR-15a-5p, hsa-miR-30a-5p, hsa-miR-126-3p, hsa-miR-148a-3p, and hsa-miR-199a-5p) using patient tumor samples. The real-time PCR results showed that these 5 miRNAs were all significantly downregulated in tumor samples from HCC patients with VI (Fig. 7a). We already knew that miRNA expression was higher in patients without VI in Fig. 7a, then we further explored the prognostic effects of these 5 miRNAs in HCC patients without VI. According to the patient's clinical follow-up information in Additional file 1: Table S1, we selected 6 cases with metastasis/relapse within 1 year and considered their prognosis to be poor. We selected 6 cases with no metastasis within 2 years and considered their prognosis good. The heatmap result showed us that the expression level of these 5 miRNAs was lower in patients with early metastasis/relapse (Fig. 7b), which was consistent with the result in Fig. 5f. However, not all 5 miRNAs were significantly different between the two groups (Additional file 1: Figure S1A).

### VI-related miRNA–mRNA regulatory network

**Fig. 8:**
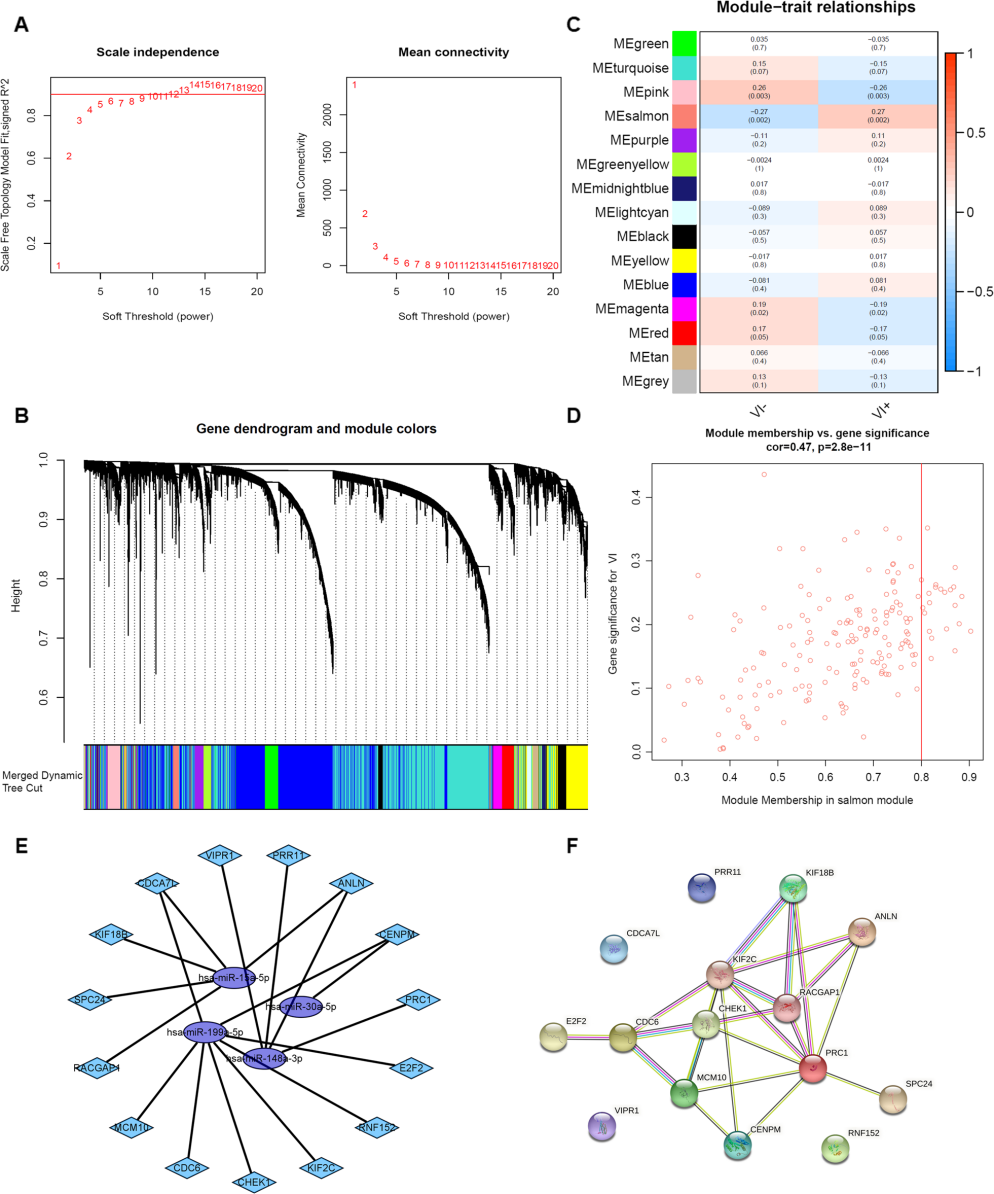
Construction of the VI-related miRNA-mRNA regulatory network. (**a**) The soft threshold determination was based on scale-free topology criterion. (**b**) Modules with different colors were assigned by the Dynamic Tree Cut algorithm. (**c**) Heatmap showed the correlation between VI and the characteristic gene value of the module. Values outside of brackets are correlation coefficients, and values inside of brackets were gene significance level. (**d**) Scatterplot showed a highly significant correlation between gene significance for VI versus module membership in the salmon module. (**e**) The miRNA-mRNA network related to VI in HCC patients. Light blue diamonds represented mRNAs; purple ellipses represented miRNAs. (**f**) Protein–protein interactions illustrated the relationships between proteins

Using dataset GSE20017, we performed WCGNA to build co-expression modules with a power β = 5 based on the scale-free topology criterion (Fig. 8a). As the results showed, 15 modules were determined (Fig. 8b, c). As illustrated in Fig. 8c, the salmon module was most positively correlated with VI. With a correlation coefficient of 0.47 and *p*-value < 0.05, the salmon module showed a highly significant correlation between gene significant for VI and module membership in this module (Fig. 8d). As we all know, miRNAs play biological effects mainly by directly targeting the 3'untranslated region (3’UTR) of mRNAs to silence them [29, 30]. According to this negative regulation mechanism, 180 genes in the salmon module were regarded as VI-related mRNAs. And then, we overlapped the predicted gene targets of 5 miRNAs with the 180 VI-related mRNAs. According to the overlapping part, the regulatory relationships were established (Fig. 8e). As shown in Fig. 8e, the purple ellipses were 4 miRNA biomarkers in the prognostic model, hsa-miR-15a-5p, hsa-miR-30a-5p, hsa-miR-148a-3p, and hsa-miR-199a-5p. The other 1 miRNA biomarker in the prognostic model had no overlapped downstream mRNAs, not included in the network. Then we used PPI to select hub genes with a high confidence filter (interaction score > 0.700), and *E2F2*, *CDC6*, *KIF2C*, *CHEK1*, *MCM10*, *CENPM*, *RACGAP1*, *KIF18B*, *ANLN*, *PRC1*, and *SPC24* were defined as hub genes (Fig. 8f). We further used the GEPIA2 online tool to verify the correlation between key genes and clinical staging. The results showed that, except for stage IV, the expression of these hub genes increased significantly in high clinical grades (Additional file 1: Figure S2A).

## Discussion

VI is a determined prognostic factor of HCC associated with adverse outcomes and included in the AJCC staging system [31, 32]. The prognostic model we constructed includes 5 VI-related miRNAs, which were all protective factors for HCC patients. Some studies have shown that their reduced expressions were related to the progression of HCC, suggesting a poor prognosis. Downregulation of hsa-miR-126-3p promoted metastasis and angiogenesis in HCC [33]. hsa-miR-148a-3p was significantly downregulated in recurrent cases [34]. hsa-miR-15a-5p could downregulate *GLI2*, and the capture of it by Circ ZNF609 released *GLI2*, thereby mediating HCC progression [35]. Some other studies found that hsa-miR-30a-5p overexpression attenuated the enhanced migrative and invasive abilities of loc339803-overexpressed HCC cells [36]. hsa-miR-199a-5p could target *SNAI1*, so LINC01133 promoted HCC progression by sponging hsa-miR-199a-5p and activating *SNAI1* [37]. Both our research and these studies showed that they could be used as biomarkers for the prognosis of HCC. Besides, some recent studies claimed that miRNAs could regulate cancer cell death and affect treatment. For example, Ming-Yao Chen et al. reported that upregulation of hsa-miR-30a-5p induced by hydroxychloroquine could modulate autophagy, apoptosis, and oxidative stress in HCC to overcome sorafenib-resistance via *TLR9*/*SOD1*/hsa-miR-30a-5p/*Beclin-1* axis [38].

Clinically, the prognostic performance of a single biomarker is often unstable and challenging to have universal applicability. Therefore, in recent years, many studies have combined multiple biomarkers to construct prognostic signatures or models. For example, Zhuolun Sun et al. constructed five autophagy-related long non-coding RNAs (lncRNAs) signature to predict bladder urothelial carcinoma patients’ OS [39]. Ruoyan Zhang et al. constructed 6 VI-related genes prognostic model to predict prognosis in HCC patients [40]. This study constructed a prognostic model using 5 VI-related miRNAs and tested its prognostic performance in the TCGA database. The prognostic model showed good predictive performance and had potential clinical application value. The subsequent multi-index ROC curves showed that the prediction accuracy of the prognostic model was better than other clinicopathological parameters. There was more information in the multi-index ROC curve of the grouped cases, that is, as can be seen in Fig. 5f, in the HCC without VI group, T-stage and AJCC stage were poor to predict the prognosis of patients, while our prognostic model was great in predicting the prognosis. The ratio of the AUC value between the prognostic model and the T stage or AJCC stage was 0.707 to 0.565 or 0.558, which was in line with our expectation. The biomarkers in our prognostic model were all factors correlated with the incidence of VI. Therefore, in theory, these 5 biomarkers should be able to predict the occurrence of VI at an early stage and then more sensitively prompt the patient's prognosis. Therefore, especially for HCC patients without VI, our prognostic model can help them assess the prognosis well, which was consistent with Fig. 7b.

Previously, a few studies have also used VI-related miRNAs to construct a prognostic model/signature for HCC patients. For example, the study of Bing Han et al. built a model that could predict VI and recurrence risk well. However, no ROC curve was plotted to verify the performance of these miRNAs in predicting OS [20]. Our model could predict the 5-year survival rates well, with an AUC of 0.709, which means that the model was valuable for managing HCC patients. The study of Sung Kyu Song et al. found three miRNAs that could predict VI well. They were hsa-miR-99a-5p, hsa-miR-100-5p, and hsa-miR-148a-3p and hsa-miR-148a-3p was also included in our model [19]. We put their miRNAs into the TCGA database for further verification and found that the prediction performance of the 5-year survival rates of these miRNAs are not as good as our model (Additional file 1: Figure S3A-C). The study of Zhuo Lin et al. constructed a prognostic model containing 16 miRNAs, one of which (hsa-miR-15a-5p) was also present in our model, and there was no overlap with the other two studies above [41]. The AUC value of this model in predicting the 5-year survival rates of patients based on the TCGA database was 0.632, which is inferior to our prognostic model. Compared with the above-mentioned similar studies, our prognostic model has certain advantages in predicting prognosis in HCC patients, which suggests the model has more promising potential clinical application value. Moreover, the prognostic nomogram combining multiple clinicopathological parameters and risk score provided an intuitive tool for clinicians to improve the OS of patients [42].

Predicting the prognosis of HCC patients is an essential direction of cancer research and the key to individualized treatment after resection. A prognostic model constructed based on a specific biological process may predict the prognosis of patients and promote the personalized and rational use of medicines. However, there were not enough therapeutic targets related to VI, so we established a miRNA-mRNA regulatory network at the end of this study, which contains a total of 4 miRNAs and 15 mRNAs. This network helps us further insight into the mechanisms of VI. The hub genes screened out by the PPI network are also potential targets for preventing or treating VI in HCC patients, which provides new ideas for the prognostic treatment of HCC patients. In the miRNA-mRNA network, decreased hsa-miR-30a prompted a high incidence of portal vein tumor thrombus (PVTT) in HCC [43], hsa-miR-199a-5p targeted and downregulated *VEGFA* to inhibit tumor angiogenesis [44]. In addition, as can be seen in Fig. 2e, f, there were many pathways for VI-related miRNAs. Among them, the relationship between beta1 integrin cell surface interactions [45], glypican pathway [46], integrin family cell surface interactions [47], and *VEGF* and *VEGFR* signaling network [48] and VI has been clearly reported. The other miRNAs and mRNAs may also regulate VI through other pathways, but this hypothesis requires more research in the future to explore the mechanisms.

Hub genes determined above may be the potential targets for preventing or treating VI in HCC patients. We speculated that the five miRNAs play a biological role mainly by directly targeting the 3’UTR of mRNAs to silence them. On this basis, we have established a network related to VI. Therefore, downregulation of miRNAs and subsequent upregulation of mRNAs in this network may lead to the occurrence or development of VI, thereby promoting tumor progression, which was consistent with Additional file 1: Figure S2A, and then prompting the poor overall survival of HCC patients. In previous studies, *CDC6* was upregulated in HCC, and its family member *CDC20* was significantly upregulated in HCC patients with vascular invasion [49]. *RACGAP1* expression was significantly correlated with vascular invasion and advanced stage in gastric cancer [50]. However, these hub genes may also be involved in the occurrence of VI in other ways, such as N4-acetylcytidine, hypomethylation, and hypermethylation [51–53]. In addition, these genes may also be involved in the overall survival of HCC patients through other biological processes. For example, the expression of *KIF2C* and *PRC1* was significantly correlated with immune infiltration cells and negatively correlated with overall survival time [54, 55]. And histone H2A ubiquitination induced by *PRC1* could repress *SLC7A11* expression and then regulate ferroptosis of cancer cells [56].

In the network, hsa-miR-15a-5p potentially target and silence *RACGAP1*, *SPC24*, *KIF18B*, *CDCA7L*, and *ANLN*; hsa-miR-30a-5p potentially target and silence *CENPM*; hsa-miR-148a-3p potentially target and silence *VIPR1*, *PRR11*, *ANLN*, and *PRC1*; hsa-miR-199a-5p potentially target and silence *CDCA7L*, *CENPM*, *E2F2*, *RNF152*, *KIF2C*, *CHEK1*, *CDC6* and *MCM10*. Silencing these genes could repress HCC progression through multiple mechanisms. For example, depletion of *CENPM* could promote apoptosis, repress cell proliferation, and inhibit cell migration and invasion. The P53 signaling pathway and cell cycle pathway were involved in its functions [57]. *KIF2C* promoted HCC cell migration, invasion, and metastasis by enhancing mTORC1 signaling pathway [58].

There are several limitations in his study. Firstly, due to the limited sample size of the TCGA database and incomplete clinical information, the prognostic model and nomogram still need to be further verified by more samples and more comprehensive clinical studies before they are applied to clinical practice. Besides, the miRNA-mRNA regulatory network needs to be confirmed by biochemical experiments such as immunohistochemistry, western blot, and luciferase assay. Then, our study mainly showed the association between the miRNA-mRNA network and the prognosis of HCC, didn’t explored the causal effects of them under the mendelian randomization framework [59–61]. Finally, since liquid biopsy has the advantages of non-invasiveness and continuous sampling during treatment, the application of blood tests for these 5 miRNAs is worth exploring.

In conclusion, our study's novel prognostic model and prognostic nomogram can accurately predict 5-year OS in HCC patients. They have promising clinical application value and can also promote precision treatment of patients after surgery to a certain extent and finally improve the survival time of HCC patients.

## Supplementary Information

**Additional file 1:** Supplementary Tables S1–S4 and Supplementary Figures S1–S3.

## Data Availability

The datasets supporting the conclusions of this article are available in the Cancer Genome Atlas (https://www.ncbi.nlm.nih.gov/geo/) and Gene Expression Omnibus (https://portal.gdc.cancer.gov/).
