# Supplementary material for: A novel prognostic model for hepatocellular carcinoma based on 5 microRNAs related to vascular invasion
Source: BMC Med Genomics. 2022 Feb 24;15:34. doi: 10.1186/s12920-022-01162-7 (PMC8867887; doi:10.1186/s12920-022-01162-7)
Supplement: Supplementary file 1 — Additional file 1: Supplementary Tables S1–S4 and Supplementary Figures S1–S3. [file 12920_2022_1162_MOESM1_ESM.docx]

**Supplementary Table S1**

| Clinical information of enrolled patients in our institution. | |
| --- | --- |
| Parameters | Patient samples (n, %) |
| Gender |  |
| Male | 21, 91.3% |
| Female | 2, 8.7% |
| VI |  |
| VI+ | 5, 21.7% |
| VI- | 18, 78.3% |
| Prognosis (patients without VI) |  |
| Metastasis/relapse within 1 year | 6, 33.3% |
| Metastasis/relapse free within 2 years | 6, 33.3% |
| Metastasis/relapse free within 1 year | 5, 27.8% |
| Unknown | 1, 5.6% |
| Prognosis (patients with VI) |  |
| Metastasis/relapse within 1 year | 4, 80.0% |
| Metastasis/relapse free within 2 years | 1, 20.0% |

**Supplementary Table S2**

| Clinical information of patients with HCC from TCGA database. | |
| --- | --- |
| Parameters | TCGA set (n, %) |
| Gender |  |
| Male | 254, 68.1% |
| Female | 119, 31.9% |
| AJCC stage |  |
| Ⅰ | 170, 49.3% |
| Ⅱ | 85, 24.6% |
| Ⅲ | 85, 24.6% |
| Ⅳ | 5, 1.4% |
| VI |  |
| VI+ | 109, 34.7% |
| VI- | 205, 65.3% |
| TNM staging system (T) |  |
| T1 | 181, 49.3% |
| T2 | 93, 25.3% |
| T3 | 80, 21.8% |
| T4 | 13, 3.5% |

**Supplementary Table S3**

Log-rank. P value of miRNAs.

| miRNA | cutoff | p.log-rank |
| --- | --- | --- |
| hsa-miR-1181 | 1.074434 | 0.074 |
| hsa-miR-639 | 0.027588 | 0.0069 |
| hsa-miR-30a-3p | 5033.653 | 0.0004 |
| hsa-miR-148a-3p | 222836.9 | < 0.0001 |
| hsa-miR-199a-5p | 1371.684 | 0.0045 |
| hsa-miR-1225-5p | 0.000829 | 0.069 |
| hsa-miR-192-5p | 160779.5 | 0.0154 |
| hsa-miR-29a-3p | 21174.38 | 0.0002 |
| hsa-miR-99a-5p | 1267.452 | 0.0001 |
| hsa-miR-194-5p | 96393.99 | < 0.0001 |
| hsa-miR-1268 | / | 0.064 |
| hsa-miR-146b-5p | / | 0.13 |
| hsa-miR-1281 | / | 0.068 |
| hsa-miR-100-5p | 10250.69 | < 0.0001 |
| hsa-miR-19b-3p | 388.9601 | 0.0154 |
| hsa-let-7g-5p | 1881.475 | 0.0076 |
| hsa-miR-106b-5p | 837.3961 | 0.071 |
| hsa-miR-143-3p | 427618.9 | 0.0263 |
| hsa-miR-27a-3p | 5415.436 | 0.0094 |
| hsa-miR-30e-5p | 33744.73 | 0.0004 |
| hsa-miR-20a-5p | 2775.431 | 0.0113 |
| hsa-miR-10a-5p | 37923.49 | 0.013 |
| hsa-miR-26a-5p | 5740.597 | < 0.0001 |
| hsa-miR-30a-5p | 23656.69 | < 0.0001 |
| hsa-miR-152-3p | 576.7459 | < 0.0001 |
| hsa-miR-199b-3p | 633.4056 | 0.0209 |
| hsa-miR-126-3p | 17955.05 | < 0.0001 |
| hsa-miR-199a-3p | 651.5894 | 0.034 |
| hsa-miR-28-5p | 1022.313 | 0.0015 |
| hsa-miR-20b-5p | 19.24175 | < 0.0001 |
| hsa-miR-21-5p | 1522038 | 0.26 |
| hsa-miR-494-3p | 0.627175 | 0.26 |
| hsa-miR-497-5p | 42.96864 | 0.0018 |
| hsa-miR-195-5p | 44.45425 | 0.0003 |
| hsa-miR-15a-5p | 694.8942 | < 0.0001 |

**Supplementary Table S4**

Detail clinical information of patients with HCC from TCGA database.

| id | sur.time | state | riskScore | risk | gender | age | stage | T | VI | TNM |
| --- | --- | --- | --- | --- | --- | --- | --- | --- | --- | --- |
| CC-A3M9-01A | 0.821918 | 1 | 6.729726 | high | MALE | 45 | Stage IIIA | T3 |  | T3N0M0 |
| CC-5259-01A | 0.684932 | 0 | 6.11449 | high | FEMALE | 60 | Stage IIIC | T4 |  | T4N0M0 |
| CC-5263-01A | 0.353425 | 1 | 5.365336 | high | MALE | 35 | Stage IIIA | T3 | VI- | T3N0M0 |
| DD-A1EJ-01A | 2.753425 | 1 | 5.34768 | high | FEMALE | 71 | Stage IIIC | T1 | VI- | T1N1M0 |
| CC-A123-01A | 0.6 | 0 | 4.612729 | high | FEMALE | 24 | Stage IIIA | T3 | VI- | T3N0M0 |
| BC-A69H-01A | 1.216438 | 0 | 4.370209 | high | MALE | 64 | Stage II | T2 | VI+ | T2NXM0 |
| CC-A7II-01A | 1.093151 | 0 | 4.358829 | high | MALE | 54 | Stage IIIA | T3 |  | T3N0M0 |
| CC-A9FU-01A | 0 | 0 | 4.291675 | high | FEMALE | 52 | Stage IIIA | T3a |  | T3aN0M0 |
| DD-AADN-01A | 2.460274 | 0 | 3.83887 | high | MALE | 59 | Stage I | T1 | VI+ | T1NXMX |
| CC-A1HT-01A | 0.276712 | 1 | 3.824743 | high | MALE | 50 | Stage IIIA | T3 |  | T3N0M0 |
| CC-A8HV-01A | 0.764384 | 1 | 3.739896 | high | FEMALE | 51 | Stage II | T2 |  | T2N0M0 |
| DD-AACG-01A | 1.284932 | 1 | 3.610203 | high | MALE | 52 | Stage II | T2 | VI+ | T2N0M0 |
| RC-A6M6-01A | 0.024658 | 0 | 3.429826 | high | MALE | 75 | Stage II | T2 | VI- | T2NXM0 |
| BC-A10Z-01A | 0.093151 | 1 | 3.302423 | high | FEMALE | 62 | Stage I | T1 | VI- | T1N0MX |
| DD-AA3A-01A | 1.123288 | 1 | 3.26842 | high | FEMALE | 81 | Stage I | T1 | VI- | T1N0MX |
| BC-A10Q-01A | 3.109589 | 1 | 3.19567 | high | FEMALE | 72 |  | T2 | VI- | T2NXMX |
| ED-A7PZ-01A | 0.016438 | 0 | 3.175837 | high | MALE | 61 | Stage II | T2 | VI+ | T2NXM0 |
| CC-A5UE-01A | 0.745205 | 1 | 3.092801 | high | MALE | 48 | Stage IIIB | T4 |  | T4N0M0 |
| 2Y-A9H2-01A | 4.742466 | 0 | 2.98668 | high | FEMALE | 64 | Stage I | T1 | VI- | T1N0MX |
| CC-A7IE-01A | 0.594521 | 1 | 2.698126 | high | MALE | 57 | Stage IIIA | T3 |  | T3N0M0 |
| DD-AAD5-01A | 3.684932 | 0 | 2.682316 | high | MALE | 54 | Stage I | T1 | VI- | T1N0M0 |
| EP-A2KB-01A | 1.632877 | 1 | 2.670075 | high | FEMALE | 46 | Stage I | T1 | VI- | T1NXMX |
| CC-5258-01A | 0.353425 | 1 | 2.662572 | high | MALE | 48 | Stage II | T2 | VI- | T2N0M0 |
| G3-AAV6-01A | 0.178082 | 1 | 2.633809 | high | FEMALE | 53 | Stage IIIA | T3a | VI+ | T3aN0M0 |
| DD-A39Y-01A | 0.468493 | 1 | 2.609089 | high | MALE | 67 | Stage I | T1 | VI- | T1NXM0 |
| CC-5260-01A | 0.238356 | 1 | 2.575412 | high | FEMALE | 61 | Stage IIIC | T4 | VI- | T4N0M0 |
| G3-A7M9-01A | 0.153425 | 1 | 2.553459 | high | MALE | 70 | Stage IIIB | T3b | VI+ | T3bNXMX |
| 2Y-A9GY-01A | 2.073973 | 1 | 2.540574 | high | FEMALE | 64 | Stage II | T2 |  | T2NXMX |
| RC-A7SH-01A | 1.282192 | 0 | 2.539458 | high | MALE | 42 | Stage II | T2 | VI+ | T2N0M0 |
| DD-AACL-01A | 0.293151 | 1 | 2.526076 | high | FEMALE | 66 | Stage I | T1 | VI- | T1N0M0 |
| DD-A1EH-01A | 4.09589 | 0 | 2.484458 | high | MALE | 23 | Stage III | T3 | VI+ | T3N0M0 |
| ED-A8O6-01A | 0.153425 | 1 | 2.484035 | high | FEMALE | 50 | Stage IIIA | T3a | VI+ | T3aN0M0 |
| BC-4073-01B | 2.326027 | 0 | 2.469782 | high | MALE | 73 | Stage IIIA | T3 | VI+ | T3N0MX |
| DD-A3A2-01A | 5.838356 | 1 | 2.406877 | high | FEMALE | 76 | Stage I | T1 | VI- | T1N0M0 |
| G3-A25T-01A | 4.254795 | 0 | 2.381406 | high | FEMALE | 45 | Stage IIIA | T3 | VI- | T3N0M0 |
| BC-A10U-01A | 2.293151 | 1 | 2.283511 | high | MALE | 69 |  | T2 |  | T2NXMX |
| G3-AAV7-01A | 0.989041 | 0 | 2.278043 | high | MALE | 38 | Stage II | T2 | VI+ | T2N0M0 |
| DD-A11C-01A | 1.813699 | 0 | 2.257055 | high | MALE | 69 | Stage I | T1 | VI- | T1N0M0 |
| DD-AADB-01A | 3.40274 | 0 | 2.250442 | high | MALE | 51 | Stage I | T1 | VI- | T1N0M0 |
| DD-A1EC-01A | 1.649315 | 0 | 2.241693 | high | FEMALE | 20 | Stage I | T1 | VI- | T1N0M0 |
| MI-A75C-01A | 0.79726 | 0 | 2.228382 | high | MALE | 64 | Stage I | T1 | VI- | T1N0M0 |
| EP-A2KA-01A | 1.717808 | 1 | 2.200742 | high | FEMALE | 52 | Stage IIIA | T3a |  | T3aNXMX |
| CC-5261-01A | 0.265753 | 1 | 2.194637 | high | MALE | 44 | Stage II | T2 | VI- | T2N0M0 |
| DD-A3A1-01A | 0.638356 | 1 | 2.149065 | high | MALE | 65 | Stage IIIA | T3b | VI+ | T3bN0M0 |
| DD-A1EI-01A | 0.50137 | 0 | 2.138558 | high | MALE | 46 | Stage I | T1 | VI- | T1N0M0 |
| DD-AACZ-01A | 0.468493 | 1 | 2.123174 | high | FEMALE | 63 | Stage I | T1 | VI+ | T1N0M0 |
| FV-A3I0-01A | 2.323288 | 0 | 2.054962 | high | FEMALE | 76 | Stage II | T2 | VI+ | T2NXM0 |
| DD-A4NR-01A | 0.024658 | 1 | 2.038031 | high | FEMALE | 85 | Stage I | T1 | VI- | T1N0M0 |
| DD-A4NQ-01A | 1.021918 | 1 | 2.034976 | high | MALE | 60 | Stage II | T2 | VI+ | T2N0M0 |
| WJ-A86L-01A | 0.945205 | 0 | 2.032252 | high | FEMALE | 68 | Stage I | T1 | VI- | T1NXMX |
| DD-AACX-01A | 0.465753 | 0 | 2.008969 | high | MALE | 66 | Stage II | T2 | VI- | T2N0M0 |
| KR-A7K7-01A | 2.605479 | 0 | 1.950554 | high | FEMALE | 61 | Stage II | T2 | VI+ | T2N0M0 |
| CC-A9FW-01A | 0.679452 | 0 | 1.942732 | high | MALE | 68 | Stage IIIA | T3 |  | T3N0M0 |
| BC-4072-01B | 4.082192 | 1 | 1.935668 | high | FEMALE | 74 | Stage IIIA | T3 | VI+ | T3N0M0 |
| DD-AAED-01A | 2.090411 | 0 | 1.934415 | high | MALE | 51 | Stage I | T1 | VI- | T1N0M0 |
| UB-A7MF-01A | 0.586301 | 1 | 1.91764 | high | MALE | 56 | Stage IIIA | T3a | VI+ | T3aNXMX |
| DD-A1EK-01A | 1.528767 | 1 | 1.916922 | high | FEMALE | 64 | Stage IVB | T4 | VI- | T4N0M1 |
| DD-AAD6-01A | 1.841096 | 0 | 1.88649 | high | MALE | 66 | Stage IIIA | T3a | VI- | T3aN0M0 |
| CC-5262-01A | 0.282192 | 1 | 1.88176 | high | MALE | 67 | Stage IIIC | T4 |  | T4N0M0 |
| G3-A25W-01A | 2.561644 | 0 | 1.880815 | high | FEMALE | 79 | Stage IIIB | T3b | VI+ | T3bN0M0 |
| DD-AACP-01A | 1.136986 | 0 | 1.840213 | high | MALE | 64 | Stage I | T1 | VI- | T1N0M0 |
| DD-AADO-01A | 1.241096 | 0 | 1.793521 | high | MALE | 55 | Stage I | T1 | VI+ | T1N0M0 |
| DD-AAE1-01A | 1.512329 | 0 | 1.789767 | high | MALE | 52 | Stage I | T1 | VI- | T1N0M0 |
| DD-AACW-01A | 3.90137 | 0 | 1.784309 | high | MALE | 43 | Stage I | T1 | VI- | T1N0M0 |
| FV-A3I1-01A | 0.676712 | 1 | 1.777774 | high | FEMALE | 81 | Stage II | T2 | VI+ | T2N0MX |
| 2Y-A9H8-01A | 1.734247 | 1 | 1.762724 | high | FEMALE | 85 |  | T1 |  | T1NXMX |
| 2Y-A9GS-01A | 1.983562 | 1 | 1.748969 | high | MALE | 58 |  | T2 |  | T2NXMX |
| DD-A3A0-01A | 2.150685 | 1 | 1.723492 | high | MALE | 70 | Stage I | T1 | VI- | T1NXM0 |
| CC-A7IG-01A | 0.819178 | 1 | 1.719422 | high | MALE | 47 | Stage II | T2 |  | T2N0M0 |
| DD-A118-01A | 9.416438 | 0 | 1.718815 | high | FEMALE | 77 | Stage II | T2 | VI- | T2N0M0 |
| FV-A23B-01A | 5.073973 | 1 | 1.709713 | high | FEMALE | 70 | Stage II | T2 |  | T2N0M0 |
| K7-AAU7-01A | 0.983562 | 0 | 1.695585 | high | MALE | 61 | Stage II | T2a | VI+ | T2aNXMX |
| ED-A97K-01A | 0.016438 | 0 | 1.692808 | high | MALE | 54 | Stage IIIA | T3a | VI+ | T3aN0M0 |
| BC-A112-01A | 0.419178 | 1 | 1.67244 | high | MALE | 80 |  | T3 | VI- | T3NXMX |
| DD-A1EA-01A | 6.616438 | 0 | 1.665032 | high | MALE | 68 | Stage II | T2 | VI+ | T2N0M0 |
| ED-A82E-01A | 1.117808 | 0 | 1.65757 | high | FEMALE | 60 | Stage IIIA | T3a | VI+ | T3aN0M0 |
| DD-A3A3-01A | 1.465753 | 1 | 1.648787 | high | MALE | 45 | Stage I | T1 | VI+ | T1N0M0 |
| BC-A10S-01A | 3.89863 | 1 | 1.632026 | high | MALE | 81 |  | T3 | VI+ | T3NXMX |
| BC-A10W-01A | 0.249315 | 1 | 1.627721 | high | MALE | 50 |  | T4 |  | T4NXMX |
| FV-A4ZP-01A | 6.810959 | 1 | 1.617907 | high | MALE | 78 | Stage IIIA | T3 | VI+ | T3NXM0 |
| BC-A8YO-01A | 1.539726 | 0 | 1.614699 | high | FEMALE | 66 | Stage IIIC | T4 | VI+ | T4N0M0 |
| BC-A10R-01A | 0.843836 | 1 | 1.60631 | high | FEMALE | 66 |  | T3 | VI+ | T3NXMX |
| BD-A2L6-01A | 3.734247 | 0 | 1.602364 | high | MALE | 69 |  | T2 | VI+ | T2NXMX |
| G3-AAV4-01A | 0.073973 | 1 | 1.592462 | high | FEMALE | 83 | Stage I | T1 | VI- | T1N0M0 |
| CC-A3MB-01A | 0.863014 | 1 | 1.575003 | high | MALE | 36 | Stage IIIA | T3 |  | T3N0M0 |
| MR-A8JO-01A | 0.90411 | 0 | 1.57388 | high | MALE | 34 | Stage I | T1 | VI- | T1N0MX |
| XR-A8TC-01A | 3.668493 | 0 | 1.572157 | high | FEMALE | 43 | Stage I | T1 | VI- | T1NXMX |
| CC-A7IJ-01A | 1.046575 | 0 | 1.566794 | high | MALE | 56 | Stage II | T2 |  | T2N0M0 |
| BC-A217-01A | 3.827397 | 1 | 1.55746 | high | FEMALE | 75 | Stage II | T2 | VI+ | T2NXM0 |
| DD-AADR-01A | 5.556164 | 0 | 1.555287 | high | MALE | 58 | Stage I | T1 | VI- | T1N0M0 |
| CC-A8HU-01A | 0.942466 | 1 | 1.54427 | high | FEMALE | 39 | Stage IIIA | T3 |  | T3N0M0 |
| WX-AA47-01A | 1.523288 | 1 | 1.539651 | high | FEMALE | 33 | Stage IIIA | T3a | VI+ | T3aNXMX |
| ES-A2HT-01A | 1.2 | 1 | 1.531548 | high | MALE | 54 | Stage I | T1 | VI- | T1NXMX |
| ZP-A9CV-01A | 2.980822 | 1 | 1.517933 | high | MALE | 59 |  | T1 | VI- | T1NXMX |
| DD-A4NA-01A | 2.761644 | 0 | 1.491986 | high | FEMALE | 67 | Stage IIIC | T2 |  | T2N1M0 |
| 2Y-A9H1-01A | 3.367123 | 1 | 1.48922 | high | MALE | 58 | Stage I | T1 |  | T1NXMX |
| RC-A7S9-01A | 1.753425 | 0 | 1.484756 | high | FEMALE | 47 | Stage I | T1 | VI- | T1N0M0 |
| 5C-A9VH-01A | 0.882192 | 0 | 1.472271 | high | MALE | 70 | Stage I | T1 | VI- | T1N0M0 |
| YA-A8S7-01A | 1.128767 | 1 | 1.47154 | high | MALE | 68 | Stage IIIA | T3a | VI- | T3aN0MX |
| DD-AAVZ-01A | 5.205479 | 0 | 1.470546 | high | MALE | 38 | Stage I | T1 | VI- | T1N0M0 |
| ED-A66Y-01A | 0.810959 | 1 | 1.463264 | high | FEMALE | 51 | Stage IIIA | T3a | VI+ | T3aN0M0 |
| RC-A6M3-01A | 0 | 0 | 1.445784 | high | MALE | 24 | Stage II | T2 | VI+ | T2N0M0 |
| ED-A459-01A | 2.493151 | 0 | 1.442773 | high | MALE | 47 | Stage II | T2 | VI+ | T2N0M0 |
| BC-A3KF-01A | 0.021918 | 0 | 1.424234 | high | FEMALE | 66 | Stage I | T1 | VI- | T1NXM0 |
| FV-A4ZQ-01A | 0.032877 | 0 | 1.419267 | high | MALE | 52 | Stage I | T1 | VI- | T1NXM0 |
| 2Y-A9H5-01A | 1.520548 | 1 | 1.411656 | high | FEMALE | 59 | Stage I | T1 | VI- | T1N0MX |
| G3-A25S-01A | 1.139726 | 1 | 1.384031 | high | MALE | 64 | Stage I | T1 | VI+ | T1N0M0 |
| NI-A4U2-01A | 4.906849 | 1 | 1.380331 | high | MALE | 71 | Stage IIIA | T3 | VI- | T3NXMX |
| CC-A8HS-01A | 0.821918 | 1 | 1.372983 | high | MALE | 18 | Stage IIIC | T3 |  | T3N1M0 |
| ED-A66X-01A | 1.112329 | 0 | 1.363144 | high | MALE | 35 | Stage IIIA | T3a | VI+ | T3aN0M0 |
| RG-A7D4-01A | 3.008219 | 0 | 1.362401 | high | MALE | 69 | Stage II | T2 |  | T2N0M0 |
| BC-A10T-01A | 2.293151 | 1 | 1.356996 | high | MALE | 76 |  | T4 | VI- | T4NXMX |
| BC-A10Y-01A | 1.947945 | 1 | 1.353008 | high | MALE | 76 |  | T4 | VI+ | T4NXMX |
| DD-AAE0-01A | 1.520548 | 0 | 1.35052 | high | FEMALE | 45 | Stage IIIA | T3a | VI- | T3aN0M0 |
| DD-AADD-01A | 3.372603 | 0 | 1.346209 | high | MALE | 51 | Stage I | T1 | VI- | T1N0M0 |
| UB-A7ME-01A | 1.331507 | 0 | 1.343357 | high | MALE | 51 | Stage I | T1 | VI- | T1NXMX |
| DD-A39X-01A | 4.641096 | 1 | 1.339375 | high | FEMALE | 78 | Stage I | T1 | VI- | T1NXM0 |
| CC-A9FS-01A | 0.578082 | 0 | 1.329207 | high | MALE | 55 | Stage II | T2 |  | T2N0M0 |
| WQ-A9G7-01A | 0.082192 | 0 | 1.329073 | high | FEMALE | 71 |  | T3a | VI- | T3aNXM0 |
| DD-A3A4-01A | 1.676712 | 1 | 1.298017 | high | MALE | 37 | Stage IIIA | T3 | VI- | T3N0M0 |
| 2Y-A9H0-01A | 10.06849 | 0 | 1.286996 | high | MALE | 49 | Stage IIIA | T3 |  | T3N0M0 |
| CC-A3MA-01A | 0.830137 | 1 | 1.282765 | high | MALE | 61 | Stage IIIA | T3 |  | T3N0M0 |
| DD-A1EF-01A | 1.079452 | 1 | 1.282014 | high | FEMALE | 57 | Stage I | T1 | VI- | T1N0M0 |
| G3-A7M6-01A | 1.731507 | 0 | 1.280162 | high | FEMALE | 60 | Stage I | T1 | VI- | T1NXMX |
| G3-A25Y-01A | 1.238356 | 1 | 1.273415 | high | FEMALE | 52 | Stage I | T1 | VI- | T1N0M0 |
| CC-A3MC-01A | 0.994521 | 0 | 1.233139 | high | MALE | 54 | Stage IIIA | T3 |  | T3N0M0 |
| DD-A119-01A | 0.610959 | 1 | 1.225859 | high | MALE | 40 | Stage IV | T3a | VI+ | T3aN0M1 |
| GJ-A3OU-01A | 2.408219 | 0 | 1.220635 | high | MALE | 59 | Stage I | T1 | VI- | T1NXMX |
| DD-A1EL-01A | 1.136986 | 1 | 1.2193 | high | MALE | 23 | Stage II | T2 | VI- | T2N0M0 |
| DD-AACQ-01A | 1.183562 | 1 | 1.213414 | high | MALE | 50 | Stage II | T2 | VI- | T2N0M0 |
| DD-AADF-01A | 0.315068 | 1 | 1.198303 | high | FEMALE | 64 | Stage I | T1 | VI- | T1N0M0 |
| DD-A1EE-01A | 0.956164 | 1 | 1.18123 | high | MALE | 73 | Stage IIIA | T3 | VI- | T3N0M0 |
| DD-AADC-01A | 1.164384 | 1 | 1.175896 | high | MALE | 53 | Stage I | T1 | VI+ | T1N0M0 |
| DD-AADQ-01A | 1.194521 | 0 | 1.174533 | high | MALE | 59 | Stage II | T2 | VI- | T2N0M0 |
| DD-A1EB-01A | 5.526027 | 0 | 1.171727 | high | FEMALE | 72 | Stage I | T1 | VI- | T1N0M0 |
| CC-5264-01A | 0.279452 | 1 | 1.16957 | high | MALE | 71 | Stage IIIA | T3 | VI- | T3N0M0 |
| G3-A7M5-01A | 1.224658 | 0 | 1.168782 | high | MALE | 76 | Stage I | T1 | VI- | T1NXMX |
| G3-A25U-01A | 4.482192 | 0 | 1.159546 | high | FEMALE | 63 | Stage I | T1 | VI- | T1N0M0 |
| CC-A8HT-01A | 0.383562 | 1 | 1.15914 | high | MALE | 74 | Stage IIIA | T3 |  | T3N0M0 |
| CC-A9FV-01A | 0 | 0 | 1.141534 | high | MALE | 57 | Stage IIIA | T3 |  | T3N0M0 |
| UB-A7MA-01A | 2.323288 | 0 | 1.127867 | high | FEMALE | 62 | Stage II | T2b | VI+ | T2bN0M0 |
| DD-A114-01A | 3.147945 | 1 | 1.123403 | high | MALE | 42 | Stage II | T2 | VI+ | T2M0 |
| DD-A4NH-01A | 2.512329 | 0 | 1.119413 | high | FEMALE | 65 | Stage IIIB | T3b | VI+ | T3bN0M0 |
| DD-A115-01A | 6.964384 | 1 | 1.109277 | high | MALE | 53 | Stage IIIA | T3 | VI- | T3N0M0 |
| DD-AADJ-01A | 2.920548 | 0 | 1.099658 | high | FEMALE | 70 | Stage I | T1 | VI- | T1N0M0 |
| BC-A110-01A | 5.79726 | 1 | 1.096468 | high | FEMALE | 51 |  | T1 | VI- | T1NXMX |
| ZP-A9CY-01A | 2.142466 | 0 | 1.093591 | high | FEMALE | 66 |  | T1 | VI- | T1NXMX |
| UB-AA0U-01A | 0.89589 | 0 | 1.090794 | high | MALE | 60 | Stage II | T2 | VI- | T2NXMX |
| G3-AAV5-01A | 0.969863 | 0 | 1.090604 | high | MALE | 67 | Stage II | T2 | VI+ | T2N0M0 |
| CC-A7IL-01A | 0.761644 | 1 | 1.090092 | high | MALE | 61 | Stage IIIA | T3 |  | T3N0M0 |
| DD-AADK-01A | 2.873973 | 0 | 1.088221 | high | FEMALE | 68 | Stage II | T2 | VI+ | T2N0M0 |
| G3-A25Z-01A | 1.794521 | 0 | 1.084288 | high | MALE | 58 | Stage I | T1 | VI- | T1N0M0 |
| 2Y-A9GV-01A | 6.936986 | 1 | 1.08075 | high | FEMALE | 54 | Stage I | T1 |  | T1NXMX |
| CC-A7IK-01A | 0.717808 | 1 | 1.080414 | high | MALE | 59 | Stage IIIA | T3 |  | T3N0M0 |
| XR-A8TD-01A | 2.821918 | 0 | 1.0781 | high | FEMALE | 49 | Stage IIIB | T3 | VI- | T3N0M0 |
| DD-A73A-01A | 1.994521 | 0 | 1.077436 | high | MALE | 71 | Stage I | T1 | VI- | T1N0M0 |
| MI-A75I-01A | 1.726027 | 0 | 1.054904 | high | MALE | 61 |  | T2 |  | T2NXMX |
| 2Y-A9GX-01A | 6.690411 | 0 | 1.052567 | high | MALE | 68 | Stage I | T1 |  | T1NXMX |
| DD-A113-01A | 6.643836 | 0 | 1.050784 | high | FEMALE | 55 | Stage II | T2 | VI+ | T2N0M0 |
| DD-AACV-01A | 4.194521 | 0 | 1.047444 | high | MALE | 53 | Stage I | T1 | VI+ | T1N0M0 |
| RC-A7SB-01A | 1.610959 | 0 | 1.040784 | high | MALE | 53 | Stage II | T2 | VI- | T2N0M0 |
| DD-A3A9-01A | 2.550685 | 1 | 1.037684 | high | FEMALE | 64 | Stage IVB | T4 | VI- | T4N0M1 |
| G3-AAUZ-01A | 1.315068 | 0 | 1.032625 | high | MALE | 48 | Stage I | T1 | VI- | T1N0M0 |
| DD-AAEK-01A | 2.923288 | 0 | 1.030362 | high | MALE | 51 | Stage II | T2 | VI- | T2N0M0 |
| DD-AAEI-01A | 4.194521 | 0 | 1.024737 | high | MALE | 72 | Stage I | T1 | VI- | T1N0M0 |
| GJ-A6C0-01A | 0.084932 | 1 | 1.021893 | high | FEMALE | 75 | Stage II | T2 | VI+ | T2NXMX |
| RC-A7SF-01A | 1.586301 | 0 | 1.021278 | high | MALE | 66 | Stage I | T1 | VI- | T1N0M0 |
| DD-A73C-01A | 1.920548 | 0 | 0.993299 | high | FEMALE | 65 | Stage IIIA | T3a | VI- | T3aN0M0 |
| LG-A6GG-01A | 1.060274 | 0 | 0.992174 | high | FEMALE | 79 | Stage II | T2 | VI+ | T2NXM0 |
| DD-AACF-01A | 1 | 1 | 0.987068 | high | MALE | 68 | Stage I | T1 | VI- | T1N0M0 |
| BC-A10X-01A | 2.109589 | 1 | 0.981777 | high | FEMALE | 52 | Stage IIIA | T3a | VI+ | T3aN0MX |
| 2Y-A9GT-01A | 4.449315 | 1 | 0.979134 | high | MALE | 51 | Stage I | T1 | VI- | T1NXMX |
| DD-A3A6-01A | 8.926027 | 1 | 0.978173 | high | FEMALE | 72 | Stage II | T2 | VI+ | T2N0M0 |
| DD-AAVW-01A | 6.347945 | 0 | 0.978033 | high | MALE | 35 | Stage I | T1 | VI- | T1N0M0 |
| 4R-AA8I-01A | 0.717808 | 1 | 0.968696 | high | MALE | 66 | Stage II | T2 | VI+ | T2NXMX |
| 2Y-A9H3-01A | 4.153425 | 0 | 0.963719 | high | MALE | 45 | Stage II | T2 | VI+ | T2NXMX |
| CC-A5UC-01A | 0.950685 | 1 | 0.961294 | high | MALE | 63 | Stage IIIA | T3 |  | T3N0M0 |
| GJ-A9DB-01A | 0.183562 | 1 | 0.954171 | high | MALE | 68 | Stage I | T1 | VI- | T1N0MX |
| DD-A11D-01A | 4.273973 | 1 | 0.953108 | high | FEMALE | 57 | Stage I | T1 | VI- | T1N0M0 |
| XR-A8TF-01A | 1.89863 | 1 | 0.952038 | high | MALE | 74 | Stage I | T1 | VI- | T1NXMX |
| BD-A3EP-01A | 1.120548 | 0 | 0.951942 | high | FEMALE | 75 | Stage I | T1 | VI- | T1N0M0 |
| G3-A25X-01A | 4.873973 | 0 | 0.946319 | high | MALE | 73 | Stage II | T2 | VI- | T2N0M0 |
| WX-AA44-01A | 1.684932 | 0 | 0.945956 | high | FEMALE | 64 | Stage I | T1 | VI- | T1NXMX |
| DD-AACH-01A | 0.534247 | 1 | 0.9344 | high | MALE | 69 | Stage II | T2 | VI+ | T2N0M0 |
| DD-A39W-01A | 2.265753 | 1 | 0.931756 | high | FEMALE | 29 | Stage III | T3 | VI- | T3N0M0 |
| DD-AAD1-01A | 1.545205 | 0 | 0.93117 | high | FEMALE | 51 | Stage I | T1 | VI- | T1N0M0 |
| ED-A7PX-01A | 0.016438 | 0 | 0.930066 | low | FEMALE | 48 | Stage II | T2 | VI+ | T2NXM0 |
| CC-A5UD-01A | 0.832877 | 1 | 0.928111 | low | MALE | 45 | Stage IIIA | T3 |  | T3N0M0 |
| DD-A73F-01A | 2.972603 | 0 | 0.925901 | low | FEMALE | 77 | Stage I | T1 | VI- | T1N0M0 |
| QA-A7B7-01A | 0.257534 | 0 | 0.92535 | low | MALE | 48 | Stage II | T2 | VI+ | T2NXMX |
| G3-A5SJ-01A | 1.912329 | 0 | 0.922491 | low | MALE | 59 | Stage I | T1 | VI- | T1NXM0 |
| DD-A39Z-01A | 1.646575 | 1 | 0.918759 | low | FEMALE | 43 | Stage II | T2 | VI- | T2NXM0 |
| DD-AACN-01A | 3.567123 | 0 | 0.917657 | low | MALE | 32 | Stage I | T1 | VI- | T1N0M0 |
| FV-A2QR-01A | 1.591781 | 1 | 0.91626 | low | MALE | 75 | Stage I | T1 |  | T1N0M0 |
| DD-A11B-01A | 0.038356 | 1 | 0.915118 | low | MALE | 73 | Stage I | T1 | VI- | T1N0M0 |
| 2Y-A9H7-01A | 3.2 | 0 | 0.91317 | low | FEMALE | 81 | Stage I | T1 | VI- | T1N0MX |
| CC-A7IF-01A | 1.778082 | 1 | 0.912977 | low | MALE | 59 | Stage IIIA | T3 |  | T3N0M0 |
| DD-AAE8-01A | 1.819178 | 0 | 0.910646 | low | MALE | 45 | Stage I | T1 | VI- | T1N0M0 |
| G3-A25V-01A | 2.356164 | 0 | 0.906608 | low | MALE | 68 | Stage I | T1 | VI- | T1N0M0 |
| DD-AAE6-01A | 0.386301 | 0 | 0.901877 | low | FEMALE | 59 | Stage I | T1 | VI+ | T1N0M0 |
| DD-AACI-01A | 4.432877 | 0 | 0.90083 | low | MALE | 69 | Stage II | T2 | VI- | T2N0M0 |
| ZS-A9CE-01A | 3.4 | 0 | 0.900209 | low | FEMALE | 79 | Stage II | T2 | VI+ | T2NXMX |
| DD-AADW-01A | 1.608219 | 0 | 0.89808 | low | MALE | 48 | Stage I | T1 | VI- | T1N0M0 |
| G3-AAV1-01A | 0.983562 | 1 | 0.896151 | low | MALE | 51 | Stage IIIC | T4 | VI+ | T4N0M0 |
| DD-A1EG-01A | 3.758904 | 1 | 0.895146 | low | MALE | 76 | Stage I | T1 | VI- | T1N0M0 |
| 2Y-A9GW-01A | 3.482192 | 1 | 0.890722 | low | MALE | 64 | Stage I | T1 |  | T1N0MX |
| ED-A7PY-01A | 1.068493 | 0 | 0.871273 | low | FEMALE | 20 | Stage II | T2 | VI+ | T2NXM0 |
| DD-AACB-01A | 6.367123 | 0 | 0.869891 | low | FEMALE | 74 | Stage I | T1 | VI+ | T1N0M0 |
| G3-A3CG-01A | 1.843836 | 0 | 0.86849 | low | MALE | 80 | Stage I | T1 | VI+ | T1N0M0 |
| 5R-AA1D-01A | 1.230137 | 0 | 0.861892 | low | FEMALE | 17 | Stage IIIA | T3a | VI+ | T3aN0M0 |
| DD-AAVU-01A | 6.032877 | 0 | 0.861457 | low | MALE | 46 | Stage II | T2 | VI+ | T2N0M0 |
| 2Y-A9GU-01A | 5.312329 | 0 | 0.860372 | low | FEMALE | 55 | Stage I | T1 |  | T1NXMX |
| ZS-A9CD-01A | 3.79726 | 1 | 0.859737 | low | MALE | 73 | Stage II | T2 | VI- | T2NXMX |
| ED-A5KG-01A | 2.339726 | 0 | 0.850233 | low | FEMALE | 60 | Stage II | T2 | VI+ | T2N0M0 |
| K7-A5RG-01A | 1.421918 | 0 | 0.848346 | low | MALE | 66 | Stage I | T1 | VI- | T1NXMX |
| DD-AACK-01A | 0.024658 | 0 | 0.847895 | low | MALE | 70 | Stage I | T1 | VI+ | T1N0M0 |
| XR-A8TE-01A | 2.534247 | 0 | 0.841422 | low | MALE | 16 | Stage IIIA | T3 | VI- | T3N0MX |
| BD-A3ER-01A | 3.054795 | 0 | 0.841364 | low | MALE | 62 | Stage II | T2 | VI- | T2NXMX |
| KR-A7K8-01A | 2.482192 | 0 | 0.837571 | low | MALE | 57 | Stage I | T1 | VI- | T1N0M0 |
| ES-A2HS-01A | 1.884932 | 1 | 0.827873 | low | MALE | 80 | Stage I | T1 | VI- | T1NXMX |
| DD-AAD2-01A | 1.80274 | 0 | 0.827414 | low | MALE | 66 | Stage I | T1 | VI+ | T1N0M0 |
| ZP-A9D1-01A | 0.057534 | 0 | 0.825014 | low | FEMALE | 56 |  | T1 | VI- | T1NXMX |
| DD-A4NG-01A | 2.19726 | 1 | 0.822765 | low | MALE | 77 | Stage IIIA | T3a | VI+ | T3aNXM0 |
| MI-A75G-01A | 1.912329 | 0 | 0.817694 | low | MALE | 63 | Stage II | T2 | VI- | T2N0M0 |
| G3-A7M8-01A | 1.178082 | 0 | 0.816787 | low | MALE | 31 | Stage I | T1 | VI- | T1NXMX |
| DD-AAVX-01A | 4.706849 | 0 | 0.816085 | low | MALE | 38 | Stage II | T2 | VI+ | T2N0M0 |
| DD-AADU-01A | 1.517808 | 0 | 0.812275 | low | MALE | 60 | Stage II | T2 | VI+ | T2N0M0 |
| DD-AACJ-01A | 5.758904 | 0 | 0.799552 | low | MALE | 75 | Stage II | T2 |  | T2N0M0 |
| DD-AAVY-01A | 5.39726 | 0 | 0.797809 | low | MALE | 56 | Stage IIIA | T3 | VI+ | T3N0M0 |
| G3-A3CH-01A | 2.136986 | 0 | 0.791017 | low | MALE | 53 | Stage IIIA | T3a | VI- | T3aN0M0 |
| DD-A73G-01A | 9.528767 | 0 | 0.790073 | low | FEMALE | 73 | Stage I | T1 | VI- | T1N0M0 |
| BC-A216-01A | 3.70137 | 0 | 0.79005 | low | FEMALE | 62 | Stage IIIA | T3 | VI- | T3NXM0 |
| LG-A9QD-01A | 1.00274 | 0 | 0.789729 | low | MALE | 68 | Stage IIIA | T3a | VI- | T3aN0M0 |
| KR-A7K2-01A | 2.271233 | 0 | 0.78688 | low | MALE | 64 | Stage I | T1 | VI- | T1N0M0 |
| 2Y-A9H4-01A | 3.978082 | 0 | 0.783401 | low | MALE | 68 | Stage I | T1 | VI- | T1N0MX |
| DD-AADA-01A | 3.378082 | 0 | 0.781351 | low | FEMALE | 66 | Stage I | T1 | VI+ | T1N0M0 |
| PD-A5DF-01A | 1.750685 | 1 | 0.779661 | low | FEMALE | 58 | Stage IIIB | T4 |  | T4N0M0 |
| DD-AADI-01A | 2.972603 | 0 | 0.776831 | low | FEMALE | 43 | Stage I | T1 | VI- | T1N0M0 |
| DD-AAVQ-01A | 7.473973 | 0 | 0.774147 | low | MALE | 38 | Stage I | T1 | VI- | T1N0M0 |
| UB-A7MD-01A | 0.142466 | 1 | 0.774122 | low | MALE | 67 | Stage I | T1 | VI+ | T1N0MX |
| UB-AA0V-01A | 0.860274 | 0 | 0.763144 | low | FEMALE | 69 |  |  | VI- | NXMX |
| DD-AADS-01A | 1.29863 | 0 | 0.760975 | low | MALE | 63 | Stage I | T1 | VI- | T1N0M0 |
| K7-A6G5-01A | 1.40274 | 0 | 0.759957 | low | MALE | 66 | Stage I | T1 | VI- | T1N0MX |
| EP-A12J-01A | 1.561644 | 0 | 0.758008 | low | MALE | 62 | Stage I | T1 | VI- | T1NXMX |
| DD-AACO-01A | 5.139726 | 0 | 0.754339 | low | MALE | 40 | Stage I | T1 | VI- | T1N0M0 |
| DD-A3A5-01A | 8.561644 | 1 | 0.75374 | low | FEMALE | 66 | Stage III | T3 | VI- | T3N0M0 |
| EP-A3RK-01A | 0.994521 | 0 | 0.753735 | low | MALE | 73 | Stage IIIA | T3a |  | T3aNXMX |
| DD-AAVS-01A | 4.994521 | 0 | 0.750736 | low | MALE | 56 | Stage I | T1 | VI- | T1N0M0 |
| 5C-A9VG-01A | 0.89863 | 0 | 0.750728 | low | MALE | 58 | Stage II | T2 | VI+ | T2N0M0 |
| ZS-A9CF-01A | 6.608219 | 0 | 0.750444 | low | MALE | 64 | Stage II | T2 | VI+ | T2NXMX |
| DD-AADL-01A | 1.742466 | 0 | 0.747842 | low | MALE | 58 | Stage I | T1 | VI+ | T1N0M0 |
| BC-A5W4-01A | 1.49863 | 1 | 0.747102 | low | MALE | 69 | Stage IIIA | T3a | VI+ | T3aNXM0 |
| DD-A3A7-01A | 1.147945 | 1 | 0.745186 | low | MALE | 67 | Stage IIIB | T3b | VI+ | T3bN0M0 |
| 3K-AAZ8-01A | 1.084932 | 0 | 0.735076 | low | MALE | 65 | Stage IIIB | T3b |  | T3bNXMX |
| UB-A7MC-01A | 1.369863 | 0 | 0.733533 | low | MALE | 59 | Stage IIIA | T3a | VI+ | T3aN0MX |
| LG-A9QC-01A | 1.164384 | 0 | 0.73239 | low | MALE | 48 | Stage I | T1 | VI+ | T1NXM0 |
| DD-AAE7-01A | 1.764384 | 0 | 0.729863 | low | MALE | 72 | Stage I | T1 | VI- | T1N0M0 |
| MI-A75E-01A | 1.389041 | 0 | 0.727408 | low | MALE | 61 | Stage IIIC | T4 | VI+ | T4N0M0 |
| EP-A3JL-01A | 0.830137 | 0 | 0.726113 | low | MALE | 76 | Stage I | T1 | VI- | T1NXMX |
| DD-A1ED-01A | 6.30411 | 0 | 0.725162 | low | MALE | 68 | Stage I | T1 | VI- | T1N0M0 |
| DD-AADM-01A | 0.032877 | 1 | 0.718325 | low | MALE | 58 | Stage II | T2 | VI- | T2N0M0 |
| G3-A5SI-01A | 2.10411 | 1 | 0.716878 | low | MALE | 44 | Stage II | T2 | VI- | T2N0M0 |
| ZP-A9D0-01A | 2.989041 | 0 | 0.71576 | low | FEMALE | 67 |  | T1 | VI- | T1NXMX |
| 2Y-A9GZ-01A | 2.323288 | 1 | 0.715643 | low | FEMALE | 82 | Stage II | T2 |  | T2NXMX |
| FV-A2QQ-01A | 1.99726 | 0 | 0.709959 | low | MALE | 80 | Stage I | T1 | VI- | T1N0MX |
| ED-A4XI-01A | 2.243836 | 0 | 0.708015 | low | MALE | 58 | Stage II | T2 | VI+ | T2N0M0 |
| DD-A116-01A | 4.443836 | 1 | 0.707416 | low | MALE | 68 | Stage IIIA | T3 |  | T3N0M0 |
| NI-A8LF-01A | 2.189041 | 0 | 0.705163 | low | MALE | 74 | Stage I | T1 | VI- | T1NXMX |
| G3-AAV3-01A | 1.128767 | 0 | 0.702837 | low | FEMALE | 58 | Stage II | T2 | VI- | T2N0M0 |
| ZP-A9D2-01A | 2.09589 | 1 | 0.701462 | low | MALE | 51 |  | T2 | VI+ | T2NXMX |
| DD-AACD-01A | 1.043836 | 1 | 0.693103 | low | MALE | 48 | Stage I | T1 | VI+ | T1N0M0 |
| RC-A6M5-01A | 0.041096 | 0 | 0.688367 | low | FEMALE | 20 | Stage IVA | T1 | VI- | T1N1M0 |
| DD-AAC9-01A | 0.950685 | 0 | 0.686284 | low | MALE | 51 | Stage I | T1 | VI- | T1N0M0 |
| DD-AAW0-01A | 5.520548 | 0 | 0.686274 | low | MALE | 54 | Stage I | T1 | VI- | T1N0M0 |
| DD-AAD0-01A | 0.375342 | 0 | 0.68503 | low | FEMALE | 73 | Stage I | T1 | VI- | T1N0M0 |
| T1-A6J8-01A | 0.063014 | 0 | 0.684586 | low | MALE | 68 |  | T1 | VI- | T1NXM0 |
| DD-AAVV-01A | 6.726027 | 0 | 0.683792 | low | MALE | 56 | Stage II | T2 | VI+ | T2N0M0 |
| G3-A3CK-01A | 1.60274 | 0 | 0.680222 | low | MALE | 61 | Stage I | T1 | VI- | T1N0M0 |
| RC-A6M4-01A | 0.060274 | 0 | 0.676992 | low | FEMALE | 74 | Stage IIIA | T3 | VI- | T3NXMX |
| DD-AACY-01A | 3.972603 | 0 | 0.676979 | low | MALE | 61 | Stage I | T1 | VI- | T1N0M0 |
| ED-A8O5-01A | 1.112329 | 0 | 0.676919 | low | FEMALE | 59 | Stage IIIA | T3a | VI+ | T3aN0M0 |
| DD-A4NB-01A | 2.709589 | 0 | 0.672277 | low | MALE | 25 | Stage I | T1 | VI- | T1N0M0 |
| DD-AAVR-01A | 6.884932 | 0 | 0.656703 | low | MALE | 44 | Stage I | T1 | VI- | T1N0M0 |
| DD-A4ND-01A | 7.523288 | 0 | 0.655068 | low | FEMALE | 56 | Stage I | T1 | VI- | T1N0M0 |
| KR-A7K0-01A | 0.178082 | 1 | 0.646922 | low | MALE | 65 | Stage I | T1 | VI- | T1N0M0 |
| BC-A3KG-01A | 1.863014 | 0 | 0.64032 | low | FEMALE | 68 | Stage II | T2 | VI+ | T2N0M0 |
| O8-A75V-01A | 1.473973 | 0 | 0.639379 | low | MALE | 54 | Stage I | T1 | VI- | T1NXMX |
| DD-AAEG-01A | 1.969863 | 0 | 0.637621 | low | FEMALE | 59 | Stage I | T1 | VI- | T1N0M0 |
| DD-AAD8-01A | 3.339726 | 0 | 0.635879 | low | FEMALE | 73 | Stage I | T1 | VI- | T1N0M0 |
| BW-A5NQ-01A | 0 | 0 | 0.629784 | low | MALE | 63 | Stage I | T1 | VI- | T1NXMX |
| DD-A4NN-01A | 2.463014 | 1 | 0.62939 | low | FEMALE | 56 | Stage I | T1 | VI- | T1N0M0 |
| DD-A4NS-01A | 6.728767 | 1 | 0.623885 | low | FEMALE | 61 | Stage I | T1 | VI- | T1N0M0 |
| ZS-A9CG-01A | 0.934247 | 0 | 0.623176 | low | MALE | 55 | Stage II | T2 | VI+ | T2NXMX |
| XR-A8TG-01A | 2.460274 | 0 | 0.621506 | low | MALE | 58 | Stage I | T1 | VI- | T1NXM0 |
| DD-AAEA-01A | 1.575342 | 0 | 0.615851 | low | MALE | 65 | Stage I | T1 | VI- | T1N0M0 |
| 2Y-A9H6-01A | 0.978082 | 0 | 0.613003 | low | FEMALE | 68 | Stage I | T1 | VI+ | T1NXMX |
| DD-AACU-01A | 4.293151 | 0 | 0.608347 | low | MALE | 59 | Stage I | T1 | VI- | T1N0M0 |
| DD-AACC-01A | 4.616438 | 1 | 0.607609 | low | MALE | 61 | Stage I | T1 | VI- | T1N0M0 |
| RC-A7SK-01A | 1.293151 | 0 | 0.599555 | low | MALE | 59 | Stage I | T1 | VI+ | T1N0M0 |
| 5C-AAPD-01A | 0.054795 | 0 | 0.597094 | low | MALE | 61 | Stage II | T2 | VI- | T2N0M0 |
| DD-A73B-01A | 0.775342 | 1 | 0.596456 | low | FEMALE | 72 | Stage I | T1 | VI- | T1N0M0 |
| DD-AADV-01A | 1.572603 | 0 | 0.595085 | low | MALE | 50 | Stage I | T1 | VI- | T1N0M0 |
| DD-AAE2-01A | 1.747945 | 0 | 0.593407 | low | MALE | 51 | Stage I | T1 | VI- | T1N0M0 |
| DD-AADE-01A | 3.293151 | 0 | 0.592659 | low | MALE | 50 | Stage I | T1 | VI+ | T1N0M0 |
| K7-A5RF-01A | 1.728767 | 0 | 0.592655 | low | MALE | 64 | Stage I | T1 |  | T1NXMX |
| WQ-AB4B-01A | 1.082192 | 0 | 0.591596 | low | MALE | 62 | Stage II | T2 | VI+ | T2NXM0 |
| ZP-A9CZ-01A | 1.934247 | 0 | 0.583343 | low | MALE | 72 |  | T1 | VI- | T1NXMX |
| DD-AACM-01A | 4.846575 | 0 | 0.582996 | low | MALE | 48 | Stage II | T2 | VI- | T2N0M0 |
| UB-A7MB-01A | 1.646575 | 0 | 0.581658 | low | MALE | 24 | Stage II | T2 | VI+ | T2NXMX |
| DD-AADY-01A | 1.520548 | 0 | 0.578799 | low | FEMALE | 55 | Stage I | T1 | VI- | T1N0M0 |
| BW-A5NO-01A | 0.054795 | 0 | 0.570787 | low | MALE | 50 | Stage IIIA | T3a | VI- | T3aNXMX |
| DD-AACT-01A | 4.279452 | 0 | 0.566922 | low | FEMALE | 69 | Stage I | T1 | VI- | T1N0M0 |
| DD-AAE3-01A | 1.550685 | 0 | 0.566385 | low | MALE | 50 | Stage I | T1 | VI- | T1N0M0 |
| EP-A2KC-01A | 0.052055 | 1 | 0.558526 | low | MALE | 62 | Stage I | T1 | VI- | T1NXMX |
| ED-A627-01A | 1.158904 | 0 | 0.557022 | low | MALE | 74 | Stage I | T1 |  | T1NXM0 |
| DD-AADP-01A | 1.254795 | 0 | 0.555362 | low | MALE | 45 | Stage I | T1 | VI+ | T1N0M0 |
| 2Y-A9HA-01A | 0.09863 | 1 | 0.553288 | low | MALE | 70 | Stage II | T2 |  | T2NXMX |
| MR-A520-01A | 0.627397 | 0 | 0.537451 | low | MALE | 58 | Stage I | T1 |  | T1NXMX |
| BW-A5NP-01A | 0 | 0 | 0.530924 | low | FEMALE | 26 | Stage IV | T2 | VI+ | T2N0M1 |
| 5R-AAAM-01A | 0.126027 | 1 | 0.529836 | low | FEMALE | 65 | Stage II | T2 | VI+ | T2N0M0 |
| G3-A5SM-01A | 1.424658 | 0 | 0.526291 | low | MALE | 58 | Stage II | T2 | VI- | T2NXM0 |
| ZS-A9CF-02A | 6.608219 | 0 | 0.525276 | low | MALE | 64 |  |  |  | T2NXMX |
| ED-A7XO-01A | 1.169863 | 0 | 0.524111 | low | MALE | 29 | Stage IIIA | T3a | VI+ | T3aN0M0 |
| ED-A7XP-01A | 1.09589 | 0 | 0.522844 | low | FEMALE | 53 | Stage II | T2 | VI+ | T2N0M0 |
| G3-A3CJ-01A | 1.627397 | 0 | 0.521781 | low | MALE | 52 | Stage II | T2 | VI+ | T2N0M0 |
| DD-AACA-02B | 6.30411 | 0 | 0.513661 | low | MALE | 65 |  |  |  | T1N0M0 |
| G3-A6UC-01A | 1.838356 | 0 | 0.513354 | low | MALE | 65 | Stage IIIB | T3b | VI+ | T3bN0M0 |
| DD-AACS-01A | 4.942466 | 0 | 0.510598 | low | MALE | 39 | Stage I | T1 | VI- | T1N0M0 |
| DD-AAEE-01A | 2.219178 | 0 | 0.503663 | low | MALE | 55 | Stage I | T1 | VI- | T1N0M0 |
| G3-A3CI-01A | 0.493151 | 0 | 0.500083 | low | MALE | 71 | Stage I | T1 | VI- | T1N0M0 |
| HP-A5MZ-01A | 0.249315 | 1 | 0.499976 | low | MALE | 78 | Stage I | T1 | VI- | T1NXM0 |
| 2Y-A9HB-01A | 0.712329 | 0 | 0.497244 | low | MALE | 66 | Stage I | T1 | VI- | T1NXMX |
| DD-AAC8-01A | 0.043836 | 1 | 0.495492 | low | MALE | 72 | Stage I | T1 | VI+ | T1N0M0 |
| G3-A7M7-01A | 0.989041 | 0 | 0.485378 | low | MALE | 65 | Stage I | T1 | VI- | T1NXMX |
| DD-A4NL-01A | 4.687671 | 0 | 0.484768 | low | MALE | 46 | Stage I | T1 | VI- | T1N0M0 |
| DD-AADG-01A | 3.136986 | 0 | 0.459671 | low | MALE | 70 | Stage IIIA | T3a | VI- | T3aN0M0 |
| HP-A5N0-01A | 2.060274 | 1 | 0.456618 | low | FEMALE | 90 |  | TX |  | TXNXM0 |
| DD-AAD3-01A | 3.547945 | 0 | 0.451936 | low | MALE | 43 | Stage I | T1 | VI- | T1N0M0 |
| FV-A495-01A | 0.00274 | 0 | 0.4492 | low | FEMALE | 51 | Stage II | T2 | VI- | T2NXM0 |
| CC-A7IH-01A | 1 | 0 | 0.43663 | low | MALE | 58 | Stage IIIA | T3 |  | T3N0M0 |
| FV-A496-01A | 0.027397 | 0 | 0.43508 | low | FEMALE | 84 | Stage I | T1 | VI- | T1NXM0 |
| DD-AAE9-01A | 1.978082 | 0 | 0.434179 | low | MALE | 69 | Stage I | T1 | VI- | T1N0M0 |
| G3-AAV0-01A | 1.30411 | 0 | 0.424386 | low | MALE | 58 | Stage I | T1 | VI- | T1N0M0 |
| G3-AAV2-01A | 1.019178 | 0 | 0.423196 | low | MALE | 50 | Stage I | T1 | VI- | T1N0M0 |
| DD-A3A8-01A | 0.030137 | 1 | 0.42158 | low | MALE | 75 | Stage II | T2 | VI- | T2N0M0 |
| DD-AACA-02A | 6.30411 | 0 | 0.409101 | low | MALE | 65 |  |  |  | T1N0M0 |
| DD-AAE4-01A | 1.665753 | 0 | 0.40053 | low | FEMALE | 49 | Stage I | T1 | VI- | T1N0M0 |
| 2Y-A9H9-01A | 1.909589 | 0 | 0.397956 | low | MALE | 70 | Stage I | T1 |  | T1N0MX |
| DD-AAW1-01A | 5.449315 | 0 | 0.39233 | low | MALE | 55 | Stage IIIA | T3 | VI+ | T3N0M0 |
| DD-A4NO-01A | 6.150685 | 0 | 0.3905 | low | MALE | 65 | Stage I | T1 | VI- | T1N0M0 |
| DD-AAEB-01A | 1.309589 | 0 | 0.389073 | low | MALE | 60 | Stage I | T1 | VI- | T1N0M0 |
| DD-A39V-01A | 1.761644 | 1 | 0.383194 | low | MALE | 77 | Stage II | T2 | VI- | T2NXM0 |
| G3-A5SK-01A | 2.038356 | 0 | 0.381971 | low | MALE | 58 | Stage I | T1 |  | T1NXM0 |
| DD-A4NV-01A | 6.569863 | 0 | 0.379861 | low | MALE | 61 | Stage IIIA | T3 | VI- | T3N0M0 |
| DD-AAEH-01A | 2.147945 | 0 | 0.37393 | low | MALE | 73 | Stage I | T1 | VI+ | T1N0M0 |
| EP-A26S-01A | 1.665753 | 0 | 0.37369 | low | MALE | 70 | Stage I | T1 | VI- | T1N0MX |
| DD-AACA-01A | 6.30411 | 0 | 0.365455 | low | MALE | 65 | Stage I | T1 | VI- | T1N0M0 |
| DD-A73E-01A | 0.120548 | 0 | 0.361053 | low | MALE | 66 | Stage I | T1 | VI- | T1N0M0 |
| WX-AA46-01A | 2.071233 | 0 | 0.350727 | low | MALE | 61 | Stage II | T2 | VI+ | T2NXMX |
| 5R-AA1C-01A | 1.424658 | 0 | 0.349917 | low | MALE | 57 | Stage II | T2 | VI+ | T2N0M0 |
| DD-A73D-01A | 1.89863 | 0 | 0.348996 | low | FEMALE | 68 | Stage II | T2 | VI+ | T2NXMX |
| DD-A4NI-01A | 2.235616 | 0 | 0.347259 | low | MALE | 67 | Stage II | T2 | VI- | T2NXM0 |
| G3-A5SL-01A | 1.70137 | 0 | 0.340264 | low | MALE | 70 | Stage II | T2 | VI+ | T2NXM0 |
| DD-AACE-01A | 5.983562 | 0 | 0.336812 | low | MALE | 62 | Stage I | T1 | VI- | T1N0M0 |
| MI-A75H-01A | 2.046575 | 0 | 0.331727 | low | MALE | 77 |  |  | VI+ | NXMX |
| DD-A4NE-01A | 1.808219 | 1 | 0.324465 | low | FEMALE | 75 | Stage IIIA | T3a | VI- | T3aN0M0 |
| DD-A4NP-01A | 9.063014 | 0 | 0.322941 | low | MALE | 32 | Stage I | T1 | VI- | T1N0M0 |
| DD-AAW3-01A | 4.473973 | 0 | 0.319821 | low | MALE | 69 | Stage I | T1 | VI- | T1N0M0 |
| DD-A4NF-01A | 2.580822 | 0 | 0.306141 | low | MALE | 72 | Stage I | T1 | VI- | T1N0M0 |
| ZP-A9D4-01A | 1.082192 | 0 | 0.286447 | low | FEMALE | 64 |  | T1 | VI- | T1NXMX |
| BC-A69I-01A | 1.060274 | 0 | 0.268791 | low | MALE | 69 | Stage I | T1 | VI- | T1N0M0 |
| DD-AAVP-01A | 7.539726 | 0 | 0.257089 | low | MALE | 48 | Stage I | T1 | VI- | T1N0M0 |
| DD-AAW2-01A | 5.082192 | 0 | 0.2516 | low | MALE | 69 | Stage I | T1 | VI- | T1N0M0 |

**Supplementary Figure S1.** RT-PCR results of the 5 miRNAs in patient tissues.


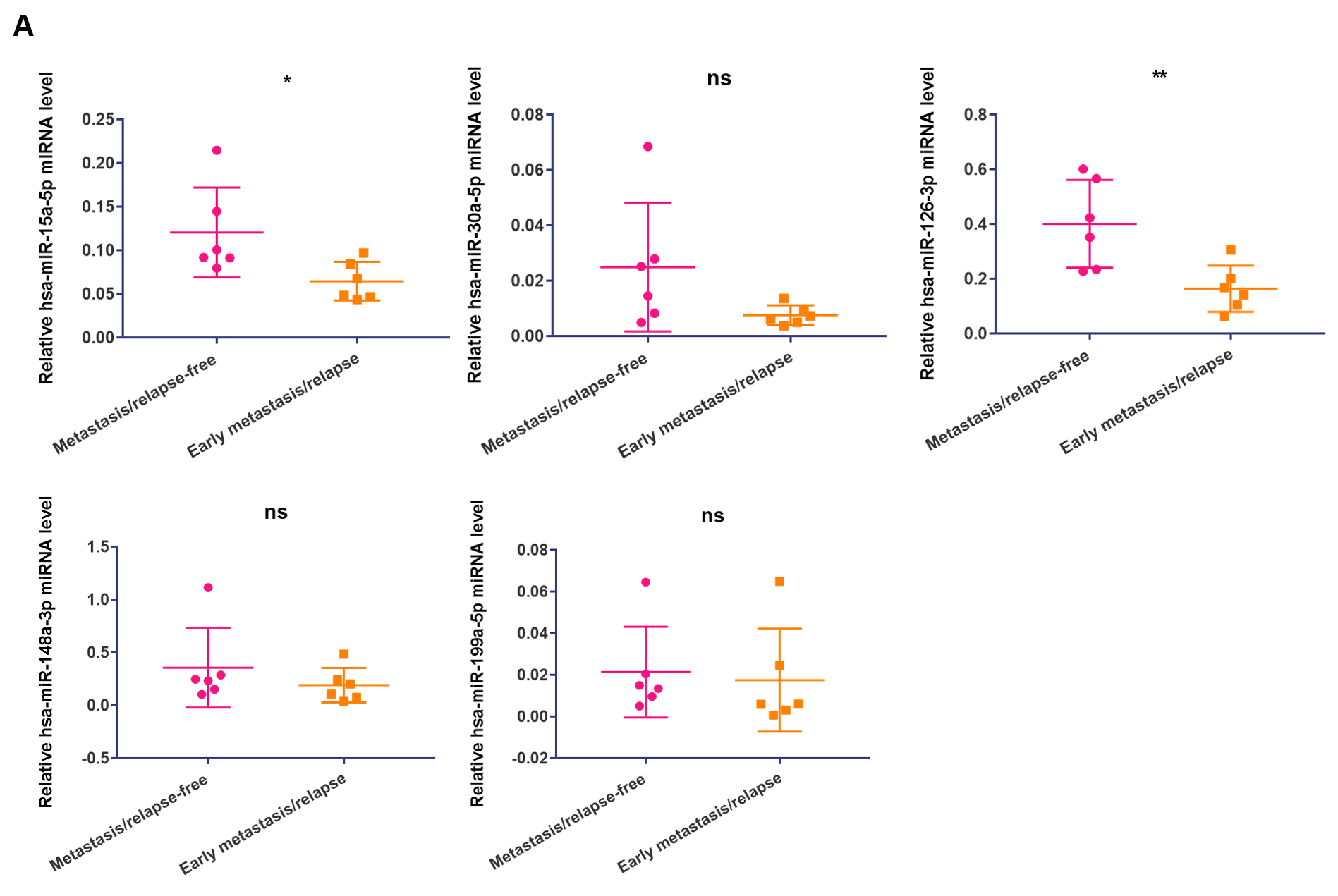


**Figure. S1.** RT-PCR results of the 5 miRNAs in patient tissues. **(A)** The expression level of 5 miRNAs in HCC patients without VI with good prognosis or poor prognosis. Early metastasis/relapse indicated a shorter survival period, and metastasis/relapse-free suggested a longer survival period.

**Supplementary Figure S2.** The correlation between hub genes and clinical staging in GEPIA 2.


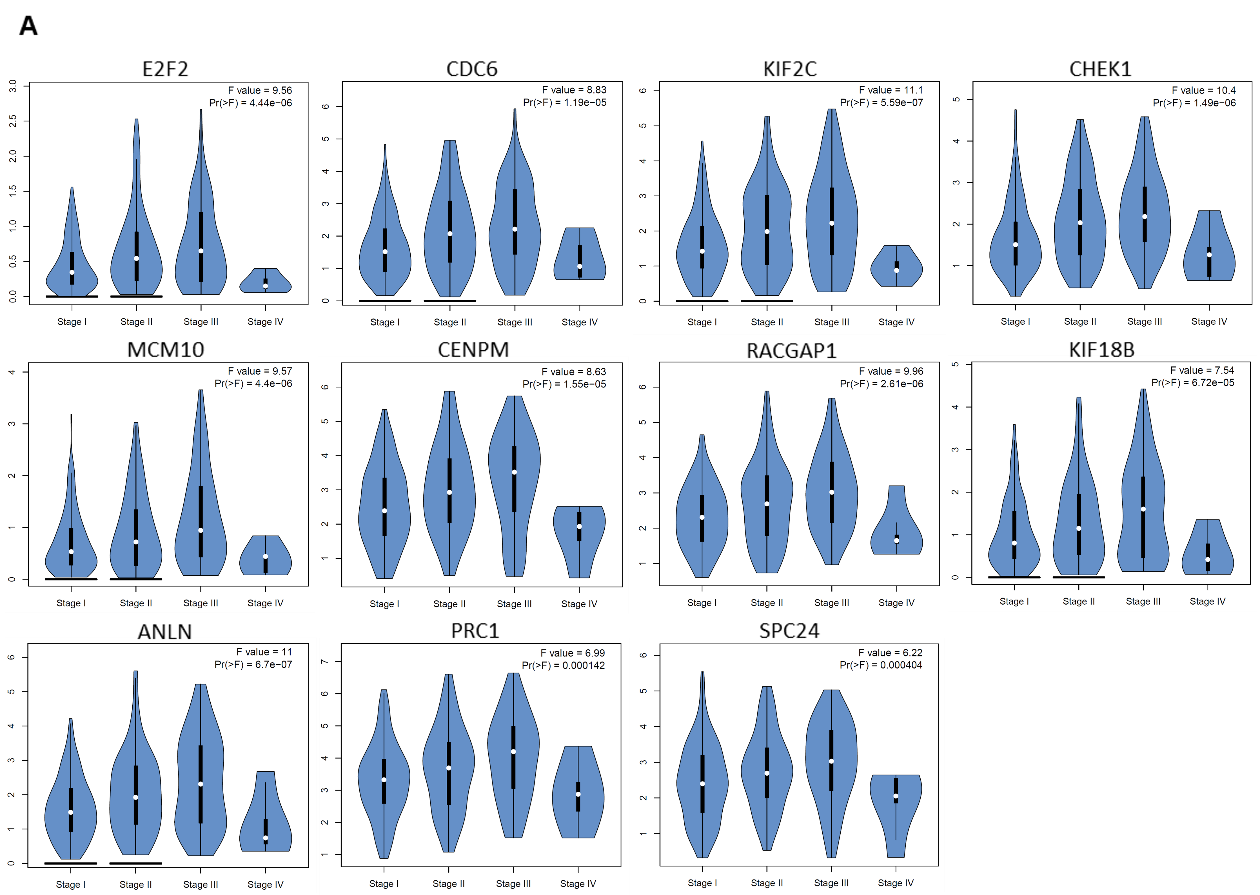


**Figure. S2.** The correlation between hub genes and clinical staging. **(A)** The violin plots showed that, except for stage Ⅳ, the expression of these hub genes increased significantly in high clinical grades.

**Supplementary Figure S3.** ROC curves of miRNAs in predicting 5-year OS of HCC patients in TCGA database.


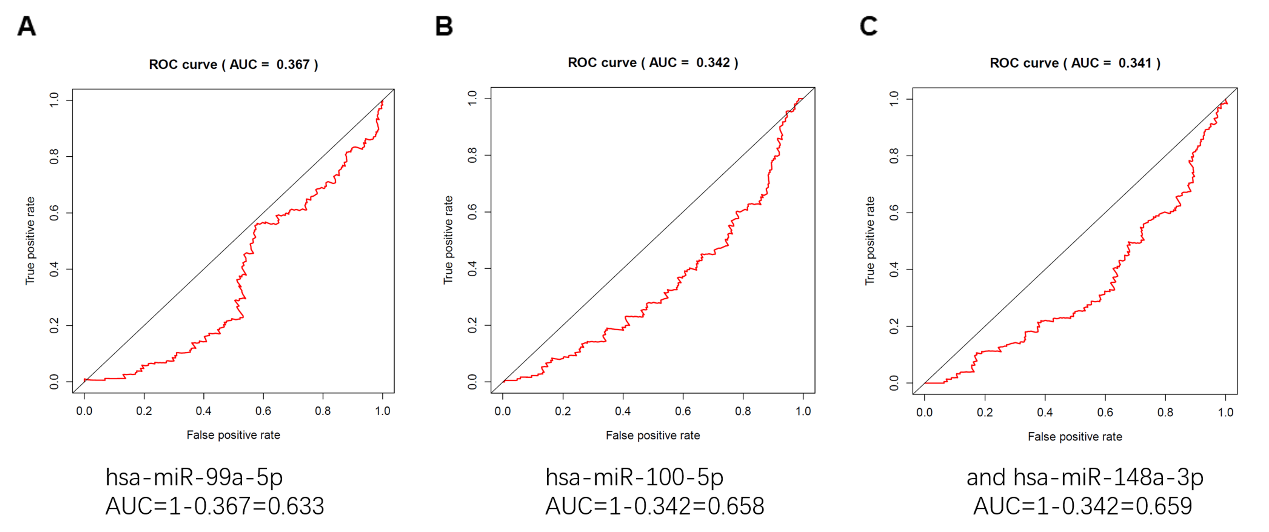


**Figure. S3.** ROC curves of miRNAs in predicting 5-year OS of HCC patients in TCGA database. **(A-C)** ROC curves of miRNAs, hsa-miR-99a-5p, hsa-miR-100-5p, and hsa-miR-148a-3p, in predicting 5-year OS of HCC patients in TCGA database.
